# Active Fabric Origami Enabled by Digital Embroidery of Magnetic Yarns

**DOI:** 10.1002/adma.202503948

**Published:** 2025-06-17

**Authors:** Haiqiong Li, Han Zhang, Xiangjun Zha, Junhong Pu

**Affiliations:** ^1^ Research Institute for Intelligent Wearable Systems The Hong Kong Polytechnic University Kowloon Hong Kong 999077 China; ^2^ School of Fashion and Textiles The Hong Kong Polytechnic University Kowloon Hong Kong 999077 China; ^3^ Department of Ultrasound Medical Research Center Affiliated Hospital of Southwest Jiaotong University The Third People's Hospital of Chengdu Chengdu Sichuan 610031 China

**Keywords:** active origami, digital embroidery, fibrous materials, magnetic textile, soft actuators

## Abstract

Active fabrics can perform deformations such as contraction, expansion, and bending when exposed to external stimuli. Origami, the ancient art of paper folding, transforms a 2D sheet into a complex 3D structure. However, integrating origami‐inspired designs into active fabrics presents significant challenges, including the large‐scale production of stimuli‐responsive yarns that can be processed using standard textile techniques to achieve intricate origami patterns with high precision and versatility. In this work, the large‐scale fabrication of magnetic yarns featuring high magnetic susceptibility, mechanical strength, and flexibility is reported, which is enabled by processing magnetic polymer composites with a series of textile engineering processes. Utilizing digital embroidery, these magnetic yarns are programmed into origami patterns with predefined yarn alignments on flexible fabrics to create various active fabric origami structures that are mechanical durable and functional consistent. These structures can reversibly transform among shapes in response to specific magnetic fields, enabling a range of functionalities such as altering surface roughness, delivering linear actuation, mimicking flower blooming, and providing switchable thermal insulation. The novel active fabric origami provides promising smart platforms across areas as diverse as smart textiles, soft robotics, wearable devices, and fashion.

## Graphical abstract

This study demonstrates a scalable textile manufacturing process that fabricates active origami fabrics (AFO) via digital embroidery of magnetic yarns. The programmable AFO exhibits reversible 2D and 3D transformations under magnetic fields, enabling functionalities such as altering surface roughness and linear actuation. These reversible deformation fabrics show promising potential for diverse applications in smart textiles and wearable devices.

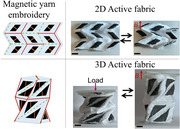

## 1 Introduction

Fabric origami, intricate crease designs such as pleating, tucking, and smocking on fabrics, not only serves as ornamentation but also enhances stretchability and fit of garments for wearers.^[^ ^1^, ^2^ ^]^ Recently, active fibrous materials that are capable of delivering reversible deformations such as contraction,^[^ ^3^, ^4^ ^]^ expansion,^[^ ^5^, ^6^ ^]^ and bending^[^ ^7^, ^8^ ^]^ in response to external stimuli are gaining increasing attention, as they open doors for smart textiles with build‐in actuation abilities. The integration of active fibrous materials with passive fabric origami creates active fabric origami (AFO) that autonomously transition between folded and deployed states.^[^ ^9^ ^]^ AFO synergize the complex structures of fabric origami with the deformability of active fibrous materials, enabling the controllable transformation of 2D fabrics into various complex 3D structures with diverse functionalities. These reconfigurable fabric systems are particularly intriguing to applications such as soft robotics,^[^ ^10^ ^]^ wearable devices,^[^ ^11^ ^]^ smart textiles,^[^ ^12^ ^]^ and aesthetics.^[^ ^13^ ^]^

AFO switch configurations by folding or bending localized regions that act as hinges, allowing connected panels to rotate. The actuation mechanisms of these hinges, enabled by active fibrous materials, fall into three categories.^[^ ^14^ ^]^ The first involves shrinkable threads that link adjacent fabric panels with a joint hinge, pulling them together by contraction.^[^ ^15^, ^16^ ^]^ However, this approach results in AFO with low structural precision, a limited folding range, and a time‐consuming fabrication process. The second strategy employs active fabric hinges, such as bilayer^[^ ^17^, ^18^ ^]^ and knitted^[^ ^19^, ^20^ ^]^ fabrics, that bend due to asymmetric deformation across their thickness in response to stimuli. These hinges typically require a large area to sufficiently rotate connected fabric panels, as they produce relatively low curvature due to the appreciable thickness. The third approach uses active fabric panels made from magnetic materials, including hard‐ and soft‐magnetic materials, which can generate directional moments to efficiently control the openness of hinges in response to magnetic fields.^[^ ^14^, ^21^ ^]^ However, hard‐magnetic fabrics suffer from restricted scalability due to the confined magnetizing space provided by magnetizers,^[^ ^22^ ^]^ whereas soft‐magnetic fiber‐like structures are structurally incompatible with fabric origami because they are usually embedded in cured polymer films.^[^ ^23^, ^24^, ^25^ ^]^

The integration of active fibrous materials with fabric origami is crucial for enabling effective and reversible transformations while maintaining the integrity of AFO.^[^ ^26^, ^27^ ^]^ Knitting liquid crystal elastomer fibers into interlocked loops can convert fiber axial contraction into localized fabric bending.^[^ ^19^, ^20^ ^]^ However, these knitted loops are prone to deformation and movement, leading to poor dimensional stability, easy detachment, and edge curling of the fabrics. Alternatively, laminating two fabric layers with mismatched expansion or contraction in response to stimuli can generate localized bending.^[^ ^28^, ^29^ ^]^ This approach often compromises durability, especially after prolonged use, due to localized shearing stress and deformation at the interface, potentially leading to delamination. Another strategy involves hand needlework, such as sewing^[^ ^16^ ^]^ and embroidery,^[^ ^30^ ^]^ to stitch active fibers or yarns into fabrics in a controlled, patterned manner, offering a high degree of integration freedom to create programmable AFO. Nevertheless, these manual procedures suffer from low precision and high labor demands. These issues can be addressed by using computerized machines for digital embroidery, allowing intricate designs, precise control, and efficient pattern fabrication with various programmable parameters, including stitch type, position, length, density, and direction.^[^ ^31^, ^32^ ^]^ Even so, high‐speed automated stitching through the needle creates significant tension and friction on the thread,^[^ ^27^, ^33^ ^]^ necessitating threads with high mechanical strength, flexibility, thin diameter, consistency, smoothness, and satisfactory elongation at break.^[^ ^34^ ^]^ Unfortunately, these demanding requirements present notable challenges for existing active magnetic fibers and yarns. As shown in Table S3 (Supporting Information), magnetic fibers/yarns reported recently exhibit insufficient breaking strength of 0.5–13 MPa, elastic modulus of 0.4–3.3 MPa, and excessive thickness, significantly limiting their compatibility with digital embroidery manufacturing processes. These material constraints emphasize the critical need for magnetic yarns capable of enabling programmable origami morphing while simultaneously meeting embroidery processing requirements.

Here, we present a magnetic yarn that can be digitally embroidered into fabrics to create AFO structures, which are remotely controllable to switch between deployed and folded configurations using magnetic fields. The magnetic yarn is composed of four twisted soft‐magnetic fibers mass‐produced by melt‐spinning and post‐drawing a polymer composite of high‐density polyethylene (HDPE) and carbonyl iron particles (CIPs). Its high magnetic susceptibility and anisotropy enable the generation of strong magnetic torque when activated by magnetic fields, causing the magnetic yarn to align its axis with the magnetic field direction. With good mechanical properties, consistency, and smoothness, the magnetic yarn facilitates automated integration into fabrics through digital embroidery, achieving high speed of over 250 stitches per minute and forming durable, performance‐stable magnetically active fabrics. This capability allows for the creation of magnetically active fabric hinges whose folding can be controlled by activating soft‐magnetic fabric panels with magnetic fields. By arranging these magnetically active fabric hinges into predefined patterns on fabrics. various AFO structures with diverse functionalities can be realized, such as a Miura‐ori AFO renders adjustable surface roughness, a tube‐shaped AFO with a Kresling pattern functions as a linear actuator, and a 3D flower AFO emulates realistic blooming. Additionally, we demonstrate an active spacer fabric enabled by AFO, capable of switching between standing and collapsed states when specific magnetic fields are applied, resulting in a 2.1‐fold change in thermal resistance quickly switched within ≈0.23 s, highlighting its potential for on‐demand personal thermal management.

## 2 Results and Discussion

### 2.1 Fabrication and Characterization of Embroiderable Magnetic Yarns

**Figure 1:**
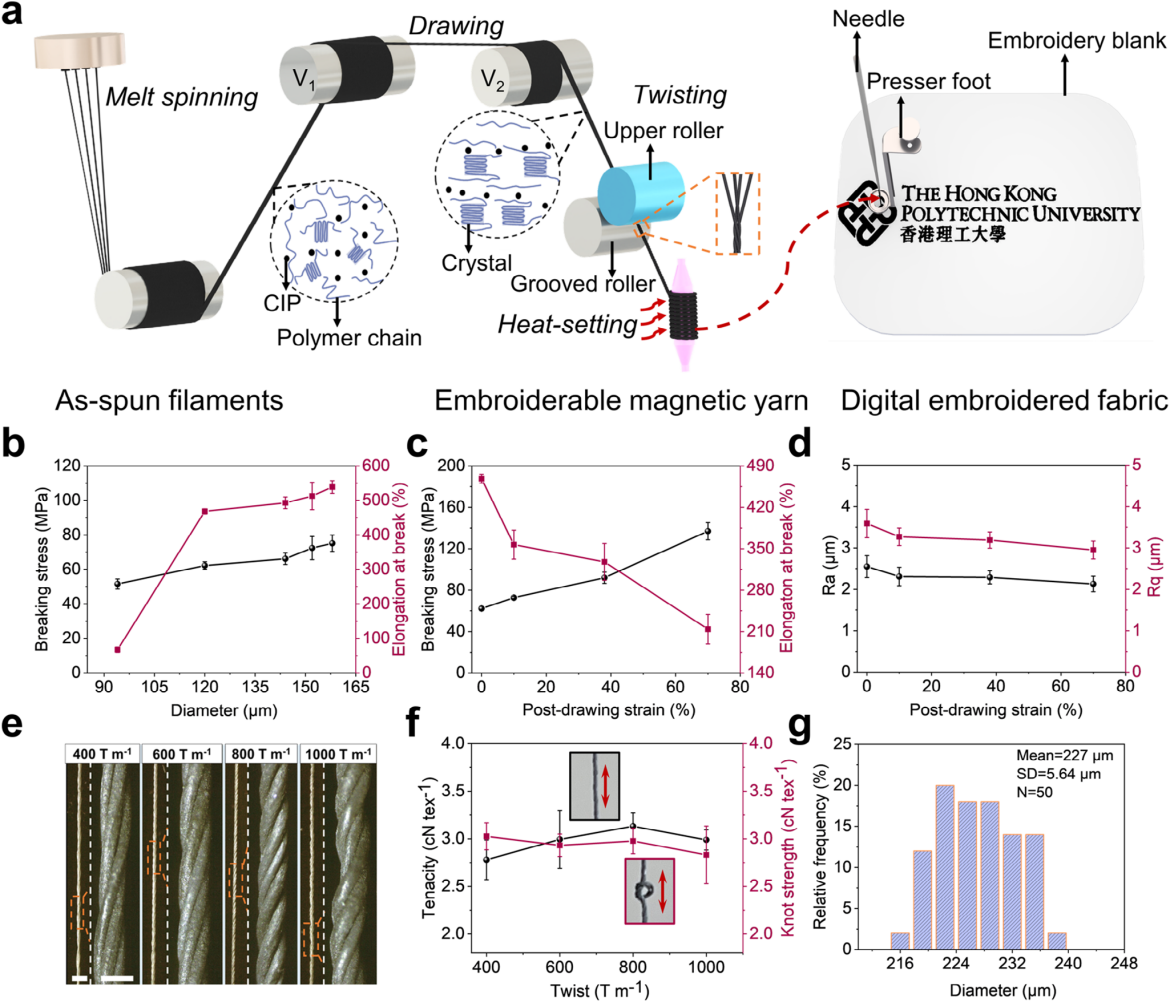
Fabrication of embroiderable magnetic yarns. a) Schematic illustration of scalable producing an embroiderable magnetic yarn and its subsequent use for creating digital embroidery designs. V_1_ and V_2_ represents the linear velocity of rotating rollers, V_1_<V_2_. b) Breaking stress and elongation at break of as‐spun magnetic filaments with different diameters. Error bars represent standard deviation (SD), n = 3 per group. c) Breaking stress and elongation at break of magnetic fibers with different post‐drawing strains. Error bars represent SD, n = 3 per group. d) Surface roughness of magnetic fibers with different post‐drawing strains. Ra and Rq represent arithmetic mean roughness and root mean square roughness, respectively. Error bars represent SD, n = 10 per group. e) Optical photographs of magnetic yarns with varying twist levels including 400, 600, 800, and 1000 turns per meter (T m^−1^). For each yarn, a global view with scale bar of 1 mm is at left showing its consistency lengthwise and a zoon‐in view with scale bar of 250 µm is at right showing its twisting structures. f) Tenacity and knot strength of magnetic yarns with different twist levels. Inserted photographs are the straight and knot structures of magnetic yarns for uniaxial tensile test with double‐head arrows indicating the stretching direction. Error bars represent SD, n = 3 per group. g) Histogram of diameter distribution of magnetic yarns with 800 T m^−1^.

An embroiderable magnetic yarn was fabricated by melt‐spinning the HDPE/CIP composite into filaments that were then post‐drawn and twisted into a yarn structure before heat‐setting, as illustrated in **Figure** **1**a. CIP, a widely used soft‐magnetic filler with high permeability and negligible remanence,^[^ ^35^ ^]^ was mixed with HDPE, a polymer commonly used for spinning high‐strength fibers such as those in fishing lines,^[^ ^36^ ^]^ to form the magnetic composite. Strong interfacial bonding between CIP and HDPE enables uniform dispersion at 70 wt.% (Note S1, Supporting Information). Through optimized spinning conditions (Table S4, Supporting Information), this high‐loading composite maintains good processability (Note S2, Supporting Information) while achieving enhanced magnetic properties, making it suitable for melt spinning. The molten composite with 70 wt.% CIP was melt‐spun into as‐spun filaments that were subsequently post‐drawn using a pair of rollers, with the back roller operating at a higher speed than the front one. Following this process, four magnetic fibers were twisted together using twisting machine. The resulting yarn, collected on a bobbin, was then heat‐setting to produce an embroiderable magnetic yarn that can be automatically stitched onto fabrics using digital embroidery machines.

The mechanical properties of as‐spun magnetic filament with varying diameters are shown in Figure 1b and Figure S7a (Supporting Information). Thinner filaments were produced by decreasing the output rate of molten composite from spinneret. As the filament diameter decreases from 158 to 94 µm, the breaking stress gradually decreases from 75.2 to 51.6 MPa, while the elongation at break decreases from 538% to 53.75%, with a significant drop to 53.75% occurring between 120 and 94 µm. This plunge is primarily due to the unsteady flow of melt extrusion at lower output rates (3 rpm). The optimization of rate to 4 rpm enabled consistent production of 120 µm diameter filaments with enhanced breaking stress and elongation at break of 62.14 MPa and 469.65%, respectively. Thus, the magnetic filament with a diameter of 120 µm was mass‐produced for post‐drawing to enhance its mechanical strength (Figure S7b, Supporting Information).

Figure 1c and Figure S8a (Supporting Information) show the mechanical properties of post‐drawn magnetic fibers with different strains obtained by adjusting velocity ratio of the front and back rollers. As the drawing strain of magnetic fibers increases from 0% to 70%, the breaking stress rises from 62 to 137 MPa showed a 121% enhancement, while the elongation at break decreases from 468% to 214%. This change is attributed to enhance polymer chain orientation achieved through post‐drawing, which is evidenced by the more pronounced relaxation of polymer chains in the amorphous regions during thermal shrinkage at 100 °C (Figure S8b, Supporting Information), and the more complete crystallization and increased crystallinity (Figure S8c,d, Supporting Information) resulting from higher post‐drawing strains. The surface of magnetic fiber becomes smoother as the post‐drawing strain increases from 0 to 70%, indicated by the decrease of Ra from 2.55 to 2.13 µm and Rq from 3.59 to 2.95 µm (Figure 1d). Increasing the strain beyond this point results in unstable fiber drawing and frequent breakage. Therefore, 70% post‐drawn magnetic fibers, which have the optimal mechanical properties, smoothest surfaces, processing stability, and enable continuous twisting, were selected for twisting into yarns for embroidery process.

We twisted four post‐drawn magnetic fibers into yarns with varying twist levels (Figure 1e). As the twist level increases from 400 to 800 T m⁻¹, the enhanced wrapping structure improves the stability against unraveling and enhances the fineness uniformity of the yarns. Meanwhile, the breaking stress of the yarns rises from 97 to 110 MPa due to improved cohesion among the fibers (Figure S9a, Supporting Information). However, further increasing the twist level to 1000 T m⁻¹ results in excessive internal stress and fiber obliquity of the yarn, which reduces both the breaking stress and strain of the yarn. The tenacity of the yarns follows a similar trend, peaking at 3.13 cN tex⁻¹ at the optimal twist level of 800 T m⁻¹ (Figure 1f). We assessed the flexibility of the yarns by subjecting knotted yarns to uniaxial tension until breakage. All yarns exhibited similar tenacities of around 3 cN tex⁻¹ in both straight and knotted formats, indicating their high flexibility and ability to withstand large strains in the knot structure. Consequently, we selected and fabricated the magnetic yarn with a twist level of 800 T m⁻¹, which demonstrated optimal mechanical properties and excellent diameter uniformity (SD/Mean = 2.48%, Figure 1g), as the embroidery yarn (Figure S9b, Supporting Information). This yarn enabled continuous embroidery and was integrated into fabrics with predefined patterns at speeds exceeding 250 stitches per minute using digital embroidery. (Figure S9c and Video S1, Supporting Information).

### 2.2 Folding Mechanisms and Properties of Magnetic Fabric Hinges

**Figure 2:**
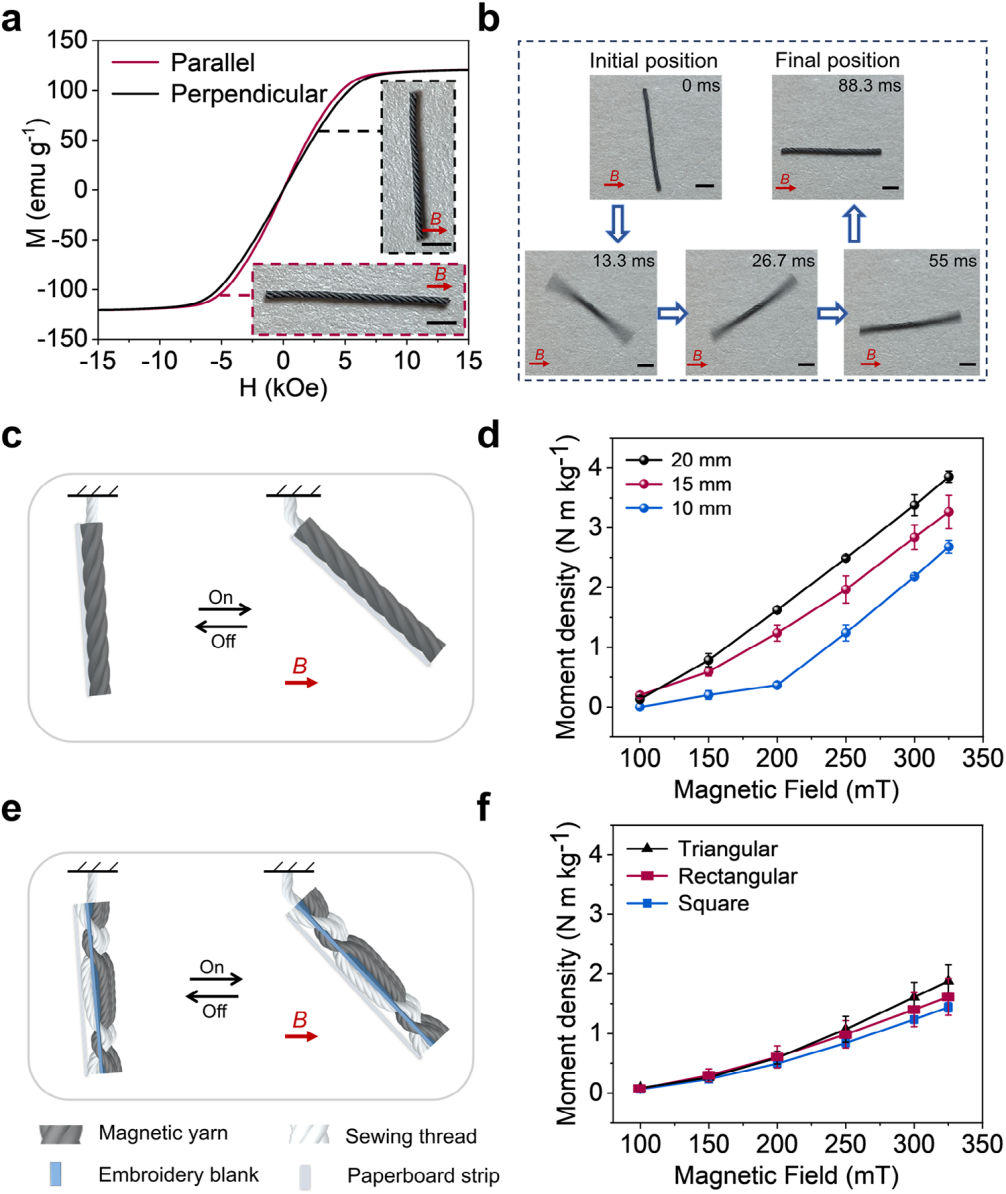
Actuation mechanisms and properties of magnetic yarns and fabrics. a) Magnetization curves of magnetic yarns magnetized parallel and perpendicular to the yarn axis. Inserts photographs show the magnetization directions. b) Sequential photographs with time stamps demonstrating the actuation of a magnetic yarn on a bench, transitioning from a non‐orthogonal to ultimately aligning orientation relative to the magnetic field direction. c) Schematic of a magnetic yarn lifting in a horizontal magnetic field. d) Relationship between moment density and magnetic field strength for magnetic yarns of varying lengths. Error bars represent SD, n = 3 per group. e) Schematic of a magnetic fabric with lock‐stitching embroidery structure lifting in a horizontal magnetic field. f) The relationship between the moment density and magnetic field strength for magnetic fabrics with triangle, rectangular, and square shapes. Error bars represent SD, n = 3 per group. Scale bars, 1 mm.

The magnetic yarn exhibits clear magnetic anisotropy, displaying different magnetization curves when magnetized parallel and perpendicular to its length, and achieves a high saturation magnetization of 120.5 emu g^−1^ (**Figure** **2**a). Figure 2b and Video S2 (Supporting Information) demonstrate that a freestanding magnetic yarn, when positioned at any angle between vertical and parallel to the magnetic field direction, experiences magnetic torque. At 0 ms, when the magnetic field is applied, a counterclockwise magnetic torque initiates rotation, reducing the angle between the yarn axis and the field direction. Driven by rotational acceleration from the torque and the moment of inertia of the yarn, it rapidly rotates past parallel alignment with the magnetic field between 13.3 and 26.7 ms. Then, the magnetic torque reverses direction, slowing the counterclockwise rotation until the yarn begins to rotate clockwise, accelerating back toward alignment. This alternating rotation persists, but the oscillation amplitude diminishes over time as energy dissipates through friction between the yarn and the bench. Ultimately, the combined action of magnetic torque and frictional damping aligns the yarn with the magnetic field, leading to magnetic energy minimization of the yarn in the magnetic field^[^ ^25^, ^37^ ^]^.

The moment densities, moment per unit mass, of magnetic yarns of varying lengths were characterized by a modified lifting test^[^ ^24^ ^]^ that measures the maximum magnetic moment output when the yarn is at 45° to the magnetic fields. The moment densities of all magnetic yarns with different lengths increase with field strength, reaching ≈4 N m kg⁻¹ at 325 mT for the 20 mm‐length yarn. At the same magnetic field strength, longer yarns exhibit higher moment densities. This is due to the bending deformation at the fixed end of the yarn, which acts as a counter‐rotation moment to resist bending, and is relatively smaller compared to the magnetic moment as the yarn length increases.

Magnetic yarns were digitally embroidered by lock‐stitching along the height of active fabric panels shaped as squares, rectangles, and triangles, all with a height of 2 cm and height‐width ratios of 1, 2, and 2, respectively (Figure S10, Supporting Information). The moment densities of these magnetic fabrics are reduced compared to single magnetic yarns (Figure 2e,f). This reduction is primarily due to the loops of the lock‐stitching magnetic yarns and non‐magnetic substrate fabrics, having zero magnetic vectors, that do not contribute to the magnetic moment in the applied magnetic field. At magnetic field strengths above 250 mT, it is observed that the moment densities of the magnetic fabrics decrease in the order of triangle, rectangle, and square. This small difference may be attributed to the decreasing aspect ratio of these shapes.

**Figure 3:**
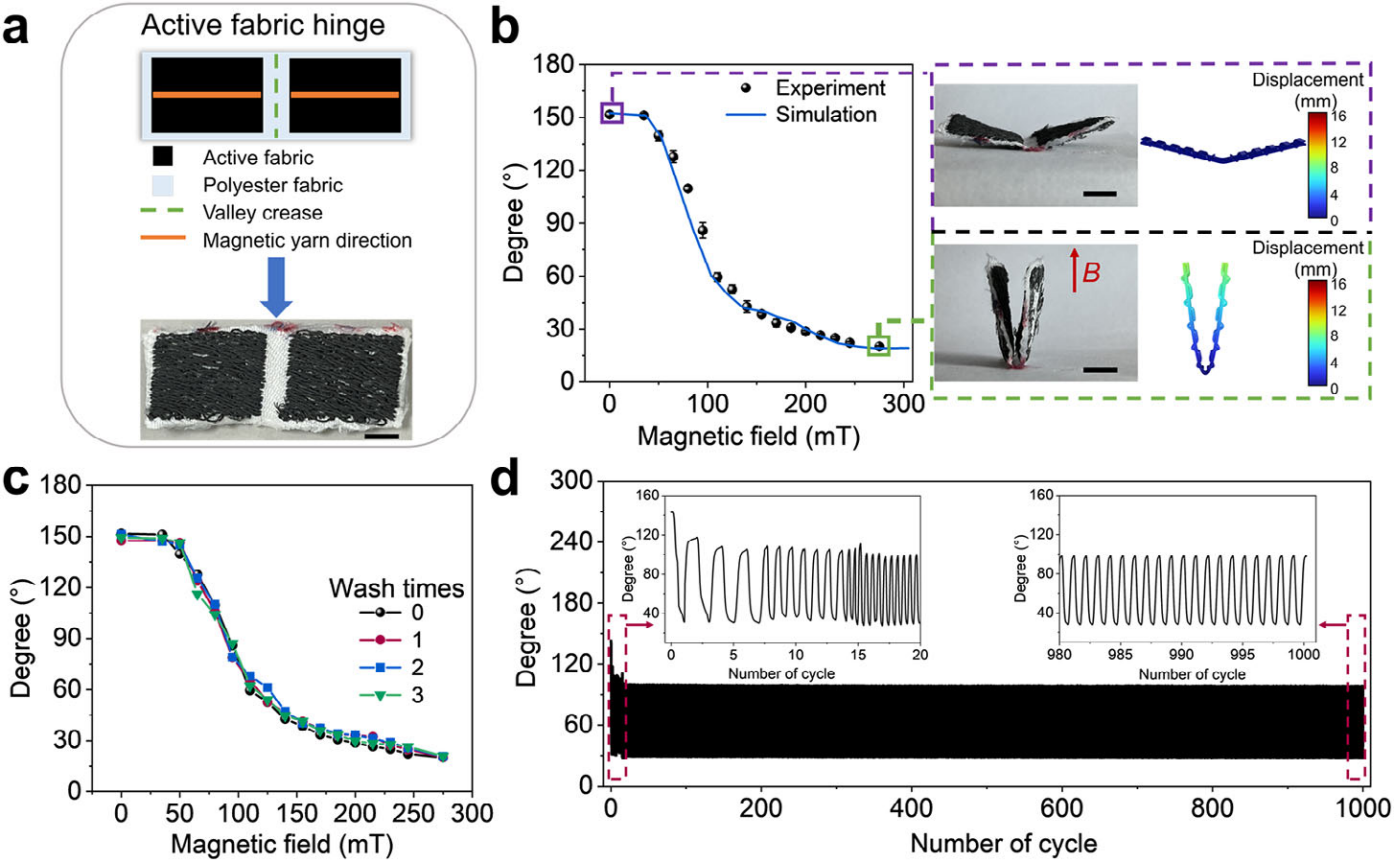
Structure and folding properties of magnetically active fabric hinge. a) Diagram of the magnetically active fabric hinge structure, showing two active fabric panels composed of magnetic yarns digitally embroidered along orange line direction on a polyester fabric with a crease. A photograph of the resulting magnetically active fabric hinge is shown below. b) Experimental and simulation results of the hinge angle as a function of magnetic field strength. Photographs and finite‐element analysis (FEA) results illustrate the folding actuation of the magnetically active fabric hinge at specific magnetic field strengths. Error bars represent SD, n = 3 per group. c) hinge angle of the magnetically active fabric hinge as a function of magnetic field strength after several washing cycles. d) Durability test of the folding actuation under a peak magnetic field strength of 150 mT over 1000 consecutive operations. Inserts show the first and last 20 cycles. Scale bars, 5 mm.

A magnetically active fabric hinge was designed and digitally embroidered, featuring two symmetrical rectangular magnetic fabrics separated by 3 mm and integrated onto a polyester fabric (**Figure** **3**a). When applying vertical magnetic fields, the collective magnetic moments generated in the magnetic fabrics induce hinge folding across a wide angular range (20° – 151°) within a narrow magnetic field strength window (0 – 275 mT), as shown in Figure 3b, showing good agreement between the experimental results and FEA prediction. In addition, the magnetically active fabric hinge retains its folding performance after multiple washes^[^ ^38^ ^]^ (Figure 3c; Figure S11, Supporting Information), and maintains stable performance during a fatigue test involving 1000 continuous actuation cycles (Figure 3d).

### 2.3 Magnetically AFO

**Figure 4:**
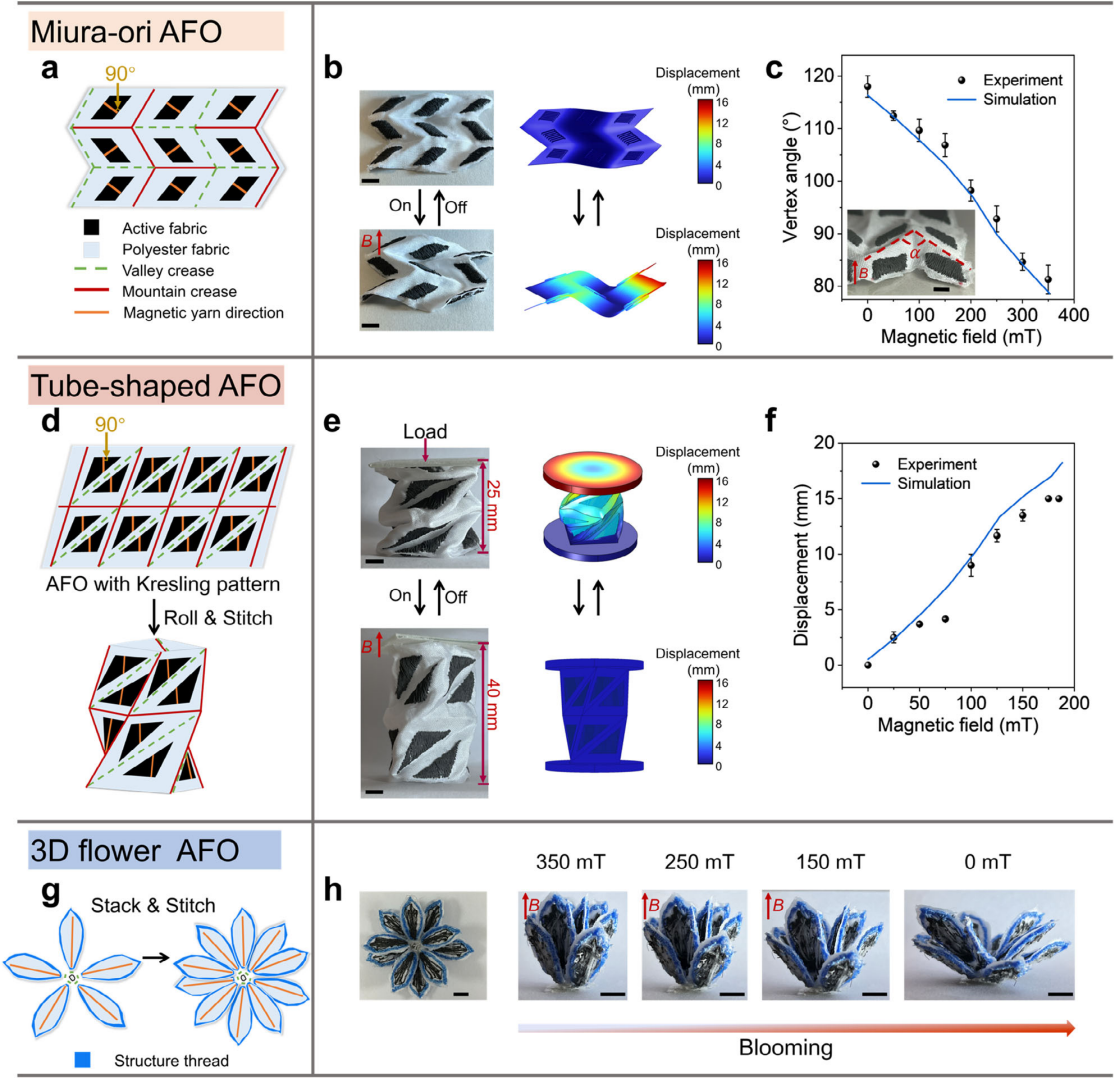
Design and behavior of magnetically AFO structures. a) Origami and magnetic pattern design of Miura‐ori AFO. b) Experiment and FEA results of the reversible deformation Miura‐ori AFO under a periodical magnetic field with a peak strength of 345 mT. c) Experimental and simulation results: vertex angle (α) of a unit cell of Miura‐ori AFO versus magnetic field strength. Error bars represent SD, n = 3 per group. d) Kresling origami and magnetic pattern design of tube‐shaped AFO. e) Experiment and FEA results of the reversible extension and contraction of the tube‐shaped AFO under a periodical magnetic field with a peak strength of 185 mT. f) Experimental and simulation results: displacement of the top surface of the tube‐shaped AFO loaded with a plastic plate (18.23 mN) versus magnetic field strength. Error bars represent SD, n = 3 per group. g) The origami and magnetic pattern design of 3D flower AFO. h) Photographs of a realistic blooming process of 3D flower AFO by gradually reducing magnetic field strength. Scale bars, 5 mm.

The magnetically active fabric hinges, exhibiting excellent folding properties and mechanical durability, enable diverse magnetically AFO with programmable deformation behaviors. We designed a Miura‐ori AFO^[^ ^39^ ^]^ composed of nine magnetically active fabric sections with specifically aligned magnetic yarns (**Figure** **4**a; Figure S12a, Supporting Information). It can reversibly transition among 3D folded configurations under out‐of‐plane magnetic fields, rendering surface roughness of 1.25 and 1.88 mm at the magnetic field strength of 0 and 345 mT, respectively (Figure 4b; Video S3, Supporting Information). Increasing the magnetic field strength enhances the moments in each active fabric section, collectively folding the Miura‐ori crease pattern and reducing the vertex angle from 118° to 81° as the field strength rises from 0 to 350 mT (Figure 4c).

We also developed a tube‐shaped AFO functioning as a linear actuator by stitching magnetic fabrics patterned with Kresling origami creases^[^ ^40^ ^]^ (Figure 4d; Figure S12b, Supporting Information). In its folded state, the tube‐shaped AFO measures 25 mm in height due to gravitational compression from its top‐mounted plate. Upon magnetic field application, moments generated in the magnetic fabric sections trigger crease unfolding, inducing rotational and axial extension of the tube (Figure 4f; Video S3, Supporting Information). Full deployment achieves a 15 mm linear displacement of the top surface.

Additionally, we demonstrated a 3D flower AFO capable of mimicking natural blooming dynamics (Figure 4g,h; Figure S12c, Supporting Information). This structure was fabricated by stacking and stitching two identical magnetic fabric layers embroidered with five petal‐shaped regions, each containing magnetic yarns radially oriented toward the center. Reducing the magnetic field strength from 350 to 0 mT drives the flower AFO from a closed bud to a fully open bloom (Figure 4h; Video S3, Supporting Information).

**Figure 5:**
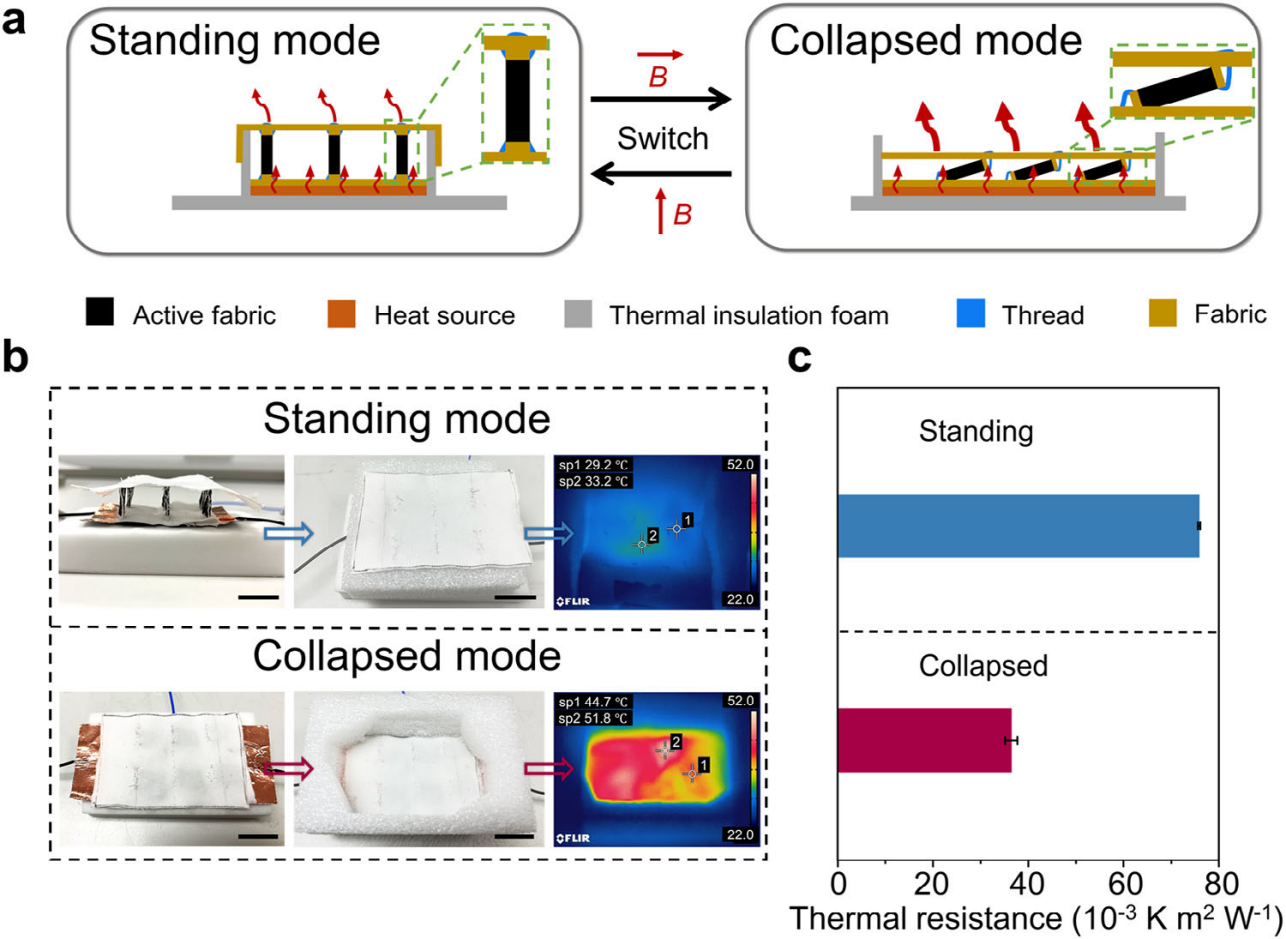
Magnetically active spacer fabric capable of switching between standing and collapsed states. a) Schematic illustration of the cross‐section structures of the active spacer fabric in standing and collapsed states. Two states with distinctive thermal insulation abilities are switchable by applying magnetic fields in horizontal and vertical directions. b) Photographs and thermal images of the standing and collapsed active spacer fabric setting on 60 °C heat sources. Scale bars, 2 cm. c) Thermal resistance of active spacer fabric in the standing and collapsed states. Error bars represent SD, n = 3 per group.

The exceptional mechanical properties of magnetically active fabrics include abrasion resistance (>1000 cycles with retained functionality, Note S3, Supporting Information) and dimensional stability (<0.1% deformation, Table S5, Supporting Information), forming the fundamental basis for complex structural designs. Expanding beyond magnetically AFO with in‐plane magnetic fabric patterns, we designed an active space fabric by integrating magnetic fabrics on substrate fabrics in an out‐of‐plane fashion. As shown in **Figure** **5**a, this structure was fabricated by whip‐stitching magnetic fabric panels between two non‐magnetic fabrics, creating friction hinges at the joints. These hinges resist pivoting motion, enabling the fabric to retain its shape indefinitely without external force. The specific magnetic actuation allows the fabric to switch between standing and collapsed states to modulate thermal insulation properties. These magnetic panels rapidly reorient under directional magnetic fields: a vertical field triggers standing mode in 0.23 s, while a horizontal field induces collapsed mode in 0.21 s (Figure S13 and Video S4, Supporting Information), with both states retaining stability without sustained magnetic field application. This rapid responsiveness of magnetic fabric surpasses that of conventional adaptive thermal management fabrics, which rely on thermal‐, humidity‐, and electrical‐activation mechanisms, and typically require several seconds to respond (Table S6, Supporting Information). Furthermore, the bistable feature eliminates the energy cost for maintaining configurations, requiring a relatively lower energy of 55.76 J for each cycle of actuation (Table S7, Supporting Information). Such a design with improved energy efficiency can enhance the portability of the whole system. For wearable applications, integrating coils could further enhance both efficiency and compactness of the system.^[^ ^41^, ^42^, ^43^ ^]^

We evaluated the thermal performance of the active spacer fabric in both states using 60 °C heat sources with adjustable power (Figure 5a,b; Figure S14, Supporting Information). The standing mode, with a thickness of 15 mm, traps air with low thermal conductivity, whereas the collapsed mode, with a thickness of 3 mm, reduces this effect. At equilibrium, the standing mode lowered the top‐layer temperature to 28 °C, compared to 45 °C for the collapsed mode, confirming tunable thermal insulation properties (Figure 5b; Figure S15, Supporting Information). Calculated thermal resistances of the standing and collapsed states are 76.05 × 10^−3^ and 36.59 × 10^−3^ K m^2^ W^−1^, respectively, yielding a 2.1‐fold adjustable window (Figure 5c). This active spacer fabric demonstrates potential for on‐demand personal thermal management, offering greater flexibility compared to current adaptive clothing.^[^ ^44^, ^45^, ^46^ ^]^ Furthermore, the active spacer fabric exhibits structural stability with water immersion resistance (Note S4, Supporting Information). It is evidently advantageous for adaptive thermal management applications, such as dynamic clothing or smart insulation textiles.

## 3 Conclusion

In summary, we have demonstrated a versatile platform for various AFO that reversibly transition between folded and deployed states in response to magnetic fields. These AFO were enabled by integrating magnetically anisotropic yarns that were mass‐produced via scalable melt‐spinning, post‐drawing, twisting and heat‐setting with fabric origami through digital embroidery. The high magnetic susceptibility and anisotropy of these yarns enable them align ability in response to magnetic fields, while the high strength, good flexibility, thin thickness, and uniform profile ensure compatibility with programmable digital embroidery techniques. By lock‐stitching these magnetic yarns into substrates, we translated their actuation capabilities into magnetic fabrics, establishing a scalable pathway for fabricating complex AFO. We studied the folding mechanisms and properties of magnetic fabric hinges as foundational building block for the magnetically AFO by FEA and experimental validation. We demonstrated AFO with distinctive structures and magnetic‐responsive behaviors including a Miura‐ori AFO that transitions among 3D folded configurations, a tube‐shaped, Kresling‐patterned AFO functioning as a linear actuator with a 15 mm stroke, and a 3D flower AFO combining aesthetic appeal with biomimetic blooming effect. The synergy between scalable magnetic yarn production and origami‐inspired textile engineering advances stimuli‐responsive fabrics, broadening the design space for smart textiles in robotics, wearable systems, and adaptive architectures. These magnetically active fabrics combine outstanding mechanical durability and performance stability, offering innovative designs for smart textiles. For instance, we demonstrate an active spacer fabric capable of rapid switching between standing and collapsed states in ≈0.23 s under magnetic field control, achieving 2.1‐fold thermal resistance tunability and maintaining a 17 °C temperature differential when exposed to a 60 °C heat source. This innovation highlights transformative potential for on‐demand personal thermal management, such as dynamic insulation in adaptive clothing.

## 4 Experimental Section

### Preparation of Embroiderable Magnetic Yarns

Carbonyl iron powder was selected for its high saturation magnetization, low coercivity, cost‐effectiveness, and oxidation resistance (with SiO₂ coating), as detailed in Table S2 (Supporting Information). Carbonyl iron powders (CIPs, SQ/BASF SE) were blended with HDPE (5000S, Lanzhou Petrochemical Company, China), which has a melt flow index of 1 g/10 min and a molecular weight (Mw) of 2.9  ×  10^5^. This mixture was processed in a high‐speed compounder with a CIP weight fraction of 70 wt.%. The HDPE/CIP mixtures were then melt‐blended in a twin‐screw extruder (Thermofisher Hot Melt Extruder Pharma 11). The temperature settings from the hopper to the die were 10, 70, 150, 250, 250, 250, 250, and 250 °C, respectively. Four different diameters of as‐spun magnetic filaments (94120, 144, 152 and 158 µm) were obtained by adjusting the speed of twin‐screw extruder. The 120 µm filament was chosen to mass production. These filaments were post‐drawn at 60 °C by adjusting the speed of the front and back rollers (AT228 Mini stretching Line, Anytester Hefei Co., Ltd Composites) to achieve post‐drawing strains of 0%, 10%, 38%, and 70%. The post‐drawing strain of a% is referred to as HDPE/CIP‐a%. The HDPE/CIP‐70% composite fibers were processed into 4‐ply yarns via a Lab Twisting machine (DW7051H, Hefei Fanyuan Instrument Co., Ltd) using “Z” twist configuration, with controlled twist levels of 400, 600, 800, and 1000 turns per meter (T m⁻¹). These yarns were heat‐set in an oven at 60 °C for 3 h to relieve residual stress and stabilize the twist configuration. Three yarns of 1 m length of magnetic yarn (800 T m⁻¹ twist) level were cut for average quality measurement, the qualities of 0.0742, 0.0618, and 0.0736 g, respectively. The average quality was calculated to be 0.06987 g. The linear density (*Nt*) of yarn is calculated by *N*_t_ =$\frac{1000G_{k}}{L}\;$, where L is yarn Length (1m); *G*_k_ is the weight of the yarn (0.06987 g). Therefore, the fineness of the magnetic yarn is 69.87 tex.

### Preparation of Magnetically AFO

Active fabrics with predefined pattern were fabricated using the magnetic yarns as top thread, an easy‐to‐pleat polyester fabric (0.15 mm thick) as the embroidery blank, and sewing thread as bottom thread. This was done with a digital embroidery machine (MDP‐S0801C (200  ×  300), Tajima Embroidery Machines Ltd) embroidering at 250 stitches per minute. Lock‐stitching was used to fabricate magnetically active fabric hinges, as well as square, rectangular, and triangular active fabric panels. However, the high stitch density of lock‐stitching makes it challenging to embroider longer stitches. Therefore, a different embroidery structure, satin‐stitching, was used to fabricate Miura‐ori active fabric, active fabric with a Kresling pattern, and flower‐shaped active fabric. After embroidery, all magnetically active fabrics were pleated according to a predesigned origami pattern at 60 °C under specific pressure for 2 h. The active fabric with a Kresling pattern was then rolled and stitched to form a tube‐shaped AFO. Two identical flower‐shaped active fabrics were stacked and stitched together to form a 3D flower AFO.

### Washability Tests

The magnetically active fabric hinge was washed for 30 min in a 100 mL beaker containing 60 mL of aqueous detergent solution (1 mg mL^−1^) with a magnetic rotator (rotator speed 500 rpm). Subsequently, the magnetically active fabric hinge sample was hand‐washed with detergent for an additional 10 min. Then the sample was dried in an oven at 60 °C for 30 min. This process was repeated three times.

### Fabrication and Characterization of Magnetically Active Spacer Fabric

A magnetically active spacer fabric (75 mm  ×  45 mm  ×  15 mm) was prepared by inlaying active fabric panels (15 mm  ×  10 mm) between two polyester fabrics using whip‐stitching. The thermal resistance of the active spacer fabric was measured using a modified standard heat‐pad method (ASTM F1868‐17). Based on the structural changes, two specifications of PE heating films were designed as heat sources: 45 mm  ×  45 mm and 75 mm  ×  45 mm. These heat sources were attached to polystyrene foam to prevent heat loss and their temperature was set to 60 °C during the experiment. The active spacer fabrics completely covered the heat sources. A precise temperature senor (thermocouple) was placed between the heat source and the bottom‐layer polyester fabric to record the “heat source temperature” (*T*_heat source_). The top‐layer polyester fabric temperature was record with a FLIR E33 IR Camera (*T*_top‐layer fabric_). Tests were conducted in a standard temperate atmosphere. The thermal resistance of active fabric (R) was calculated as $R=\frac{(T_{\mathrm{heat}\;\mathrm{source}}-T_{\mathrm{top}-\mathrm{layer}\;\mathrm{fabric}})A}{P}$. Here, A represents the area of the heat source, and P represents the power of the heat source.^[^ ^47^ ^]^

### Characterizations

Morphological observation of magnetic fibers was conducted using a scanning electron microscopy (SEM, Tescan Vega3, USA). Optical micrographs of magnetic yarns were obtained using a light stereomicroscope (Leica M165 C). The tensile properties of the samples were tested using a universal testing machine (Model 5566, Instron, USA). The surface roughness of magnetic yarns was measured using a 3D laser scanning microscope (VK‐X200, Keyence, Japan). The crystalline properties of post‐drawing magnetic fibers were determined using a differential scanning calorimetry (DSC3, Mettler Toledo Co., Switzerland). Magnetic properties were measured using the vibrating sample magnetometer (VSM, Lake Shore, Model 7404, USA).

## Conflict of Interest

The authors declare no conflict of interest.

## Author Contributions

J.P. conceived the research, obtained funding, and supervised the research. H.L. and H.Z. fabricated the magnetic yarns, embroidery items and devices. H.L., H.Z. and X.Z. carried out the experiments, simulation, and characterizations. H.L., H.Z, X.Z. and J.P. analyzed and interpreted the experimental results. H.L. and H.Z. created the figures and videos. H.L. and J.P. composed the manuscript. All authors reviewed and edited the manuscript.

## Supporting information



Supporting Information

Supplemental Video 1

Supplemental Video 2

Supplemental Video 3

Supplemental Video 4

## Data Availability

The data that support the findings of this study are available from the corresponding author upon reasonable request.
