# Supplementary material for: Active Fabric Origami Enabled by Digital Embroidery of Magnetic Yarns
Source: Adv Mater. 2025 Jun 17;37(48):2503948. doi: 10.1002/adma.202503948 (PMC12676065; doi:10.1002/adma.202503948)
Supplement: Supplementary file 1 — Supporting Information [file ADMA-37-2503948-s001.docx]

# **Supporting Information**

**Active fabric origami enabled by digital embroidery of magnetic yarns**

Haiqiong Li, Han Zhang, Xiangjun Zha, and Junhong Pu^*^

*Correspondence: [junhong.pu@polyu.edu.hk](mailto:junhong.pu@polyu.edu.hk)

**This PDF file includes:**

**Supplementary Methods**

**Supplementary Note**

**Supplementary Tables S1-S7**

**Supplementary Videos S1-S4**

# **Supplementary Methods**

**Evenness testing:** For evenness testing of magnetic yarns, cut ten yarns of 1 m length evenly from a yarn (10 m in length). Determine the diameter of each yarn of 1 m length by measuring the diameters at 5 different points and calculating their average. Calculate the coefficient of variance: CV (%)$=\frac{SD}{Mean}$ , where SD represents the standard deviation of yarn diameter, Mean represents the mean of yarn diameter.

**Differential Scanning Calorimetry (DSC):** DSC tests were conducted using a Mettler Toledo DSC3 under a nitrogen atmosphere. We characterized the melting behavior of four samples of HDPE/CIP-0%, HDPE/CIP-10%, HDPE/CIP-38% and HDPE/CIP-70% fibers. Each sample weighed approximately 1.5 mg. The temperature was set from 50 to 180 °C, with a heating rate of 10 °C/min to record the endothermic curves. The melting enthalpy (${\Delta H}_{m}$) is determined by integrating the heat flow between 100 to 140 ℃. For fully crystalline HDPE, the melting enthalpy (${\Delta H}_{m}^{0}$) is 293 J/g^[1]^. The crystallinity degree (𝑥) of testing samples was calculated by 𝑥 $(\%)=\frac{{\Delta H}_{m}}{(1-\chi) {\Delta H}_{m}^{0}}$ , where 𝑥 represents the CIPs content in the composite.

**Magnetization characterization****:** Magnetic measurements were conducted at room temperature using a Vibrating Sample Magnetometer (LakeShore 7404) with a sweep rate of 25-30 Oe/s for external magnetic fields ranging from -10 to 10 kOe. Magnetic yarns were tested axial and radial magnetization curves.

**3D laser scanning microscope****:** Surface roughness was measured using a laser confocal microscope (3D Laser Scanning Microscope, VK-X200). Measurements were conducted using a 50$\times$ objective, with a scanning area of 4$\times$40 µm^2^ at 10 random positions on each specimen, resulting in the acquisition of the average roughness parameters^[2]^.

**Abrasion test****:** The abrasion resistance test was conducted on a Martindale Abrasion and Pilling Tester (M235/8) following the ISO 12947-2:2016 standard, where each sample was cut into 44-mm-diameter circles, and then subjected to 1,000 abrasion cycles under 9 kPa pressure at 47.5 rpm along a Lissajous motion path, with subsequent observation of the sample surface condition and subsequent testing of the corresponding actuation performance.

**Moment density of** **magnetic yarns and fabrics:** To measure the moment output of magnetic yarn with a length of *L*_yarn_ and a weight of 𝑚_𝑦𝑎𝑟𝑛_, a thin paperboard strip, weighting 𝑚_𝑝_, was attached on the entire magnetic yarn. The paperboard strip was used to keep the yarn straight, enabling precise moment measurements. The magnetic field strength was set to specific values (100, 150, 200, 250, 325 mT), and the cantilevered yarn was lifted until the midpoint was blocked perpendicular to the force gauge. At this point, the yarn formed a 45° angle with the vertical direction. The force 𝐹_𝑏𝑙𝑐𝑜𝑘_ required to block the midpoint was recorded using a force gauge. The output moment per unit mass of the yarn can be calculated with equation: 𝑀_𝑝𝑚_=[(𝑚_𝑝_/𝑚_𝑦𝑎𝑟𝑛_+1) 𝑔sin45°+𝐹_𝑏𝑙𝑐𝑜𝑘_] 𝐿 /2. The output moment per unit mass of active fabric panels was measured in a similar way, with the height to width of square active fabric panels being 20 mm$\times$20 mm, the rectangular active fabric panels being 20 mm $\times$10 mm, and the triangular active fabric panels being 20 mm$\times$10 mm.

**Finite-Element Analysis of** **magnetically AFOs:** Finite-element analysis was conducted using the commercial simulation software COMSOL Multiphysics 6.2 to simulate the shape morphing process of magnetically AFOs: magnetically active fabric hinge, Miura-ori AFO and tube-shaped AFO with a Kresling pattern.

**Magnetically active fabric hinge:** We developed a force-magnetic coupling model (Figure S5) to analyze magnetically active fabric hinge. The external sphere is used to simulate the air domain, whose radius is 50 mm. The geometric dimensions of the finite element model were based on the magnetically active fabric hinge with dimensions of 15 mm$\times$10 mm$\times$0.285 mm (Figure S6a). A magnetic yarn with a radius of 0.32 mm was embroidered onto polyester fabric using lock-stitching to introduce magnetic actuation. The initial angle between the polyester fabric panel and the horizontal plane was measured as 13.49°. The material properties of the polyester fabric and magnetic yarn are shown in Table S1. A uniform magnetic field generated by two Helmholtz coil (radius is 30 mm) was applied to induce deformation of the polyester fabric panels through magnetic field forces. The magnetic field strength is controlled by electric current, and the structural deformation is calculated at 560 mA, 730 mA, 1.45 A, 1.69 A, 2.11 A, and 2.87 A. The hinge was defined along the crease line, which is the center of two polyester fabric panels with a gap of 3 mm. This crease was further constrained by locking its x, y, z translational degrees of freedom to simulate realistic folding behavior.

**Miura-ori AFO:** The simulation structure of the Miura-ori AFO is composed of several Miura units, each of which consists of four equal parallelograms (Figure S6b). During the simulation process, a model of the Miura-ori AFO was established using the same materials as the magnetically active fabric hinge model mentioned above. The uniform magnetic field generated by Helmholtz coil is also used in this model. To simulate motion constraints during the folding process, a displacement constraint in the x-direction was applied along the fold direction. The magnetic field intensity starts at 0 and gradually increases in increments of 50 mT up to 350 mT, thereby achieving the deformation process of the structure. The simulation accurately captures the stress concentration areas and the overall deformation trend of the fabric during folding, which align with experiments result.

**Tube-shaped AFO with a Kresling pattern:** The overall structure of the simulation tube-shaped AFO consists of three components: two flat planes and fabric with a Kresling pattern (Figure S6c). The bottom plane serves as a supporting platform with fixed boundary conditions applied to ensure stability throughout the loading and deformation process. The top plane is allowed to move only in the vertical direction and applies compressive loading to the tube-shaped AFO structure. A compression rate of 1 mm/s is set, with a displacement of 25 mm, which is defined as the initial fold state. After reaching the initial fold state, a uniform magnetic field is applied to the structure. Under the influence of the magnetic force, the structure gradually deploys with a displacement of 40 mm. In the tube-shaped AFO, the axial motion is coupled with torsional motion due to its unique origami patterns, and this coupling plays a critical role in the deformation characteristics of the structure. To accurately simulate real-world conditions, a general contact friction coefficient of 0.3 is applied in the model to account for the effect of surface friction between the components on the deformation behavior. The simulation successfully reproduces the deformation patterns observed in the experiment, particularly highlighting stress concentration along the creases in the stress distribution map, which aligns closely with the actual deformation regions seen in the experiment.

# **Supplementary Note**

**SEM and DSC of HDPE and composites**

**Note S1**: The cross-sectional SEM images of composites with different CIP contents (Figure S1a) shows the uniform dispersion of CIPs in the HDPE matrix without any aggregation and good wetting of HDPE on CIP surface. Figure S1b shows DSC tests on HDPE, bulk composite, and composite fiber, indicating that the addition of CIP enhances crystallinity, with the composite and composite fiber reaching 68.26% and 67.02%, respectively compared to 60.45% of pure HDPE. These results reveal the effective nucleation promotion by CIP during crystallization, confirming the interfacial interaction between CIP and polymer chains^[3, 4]^.


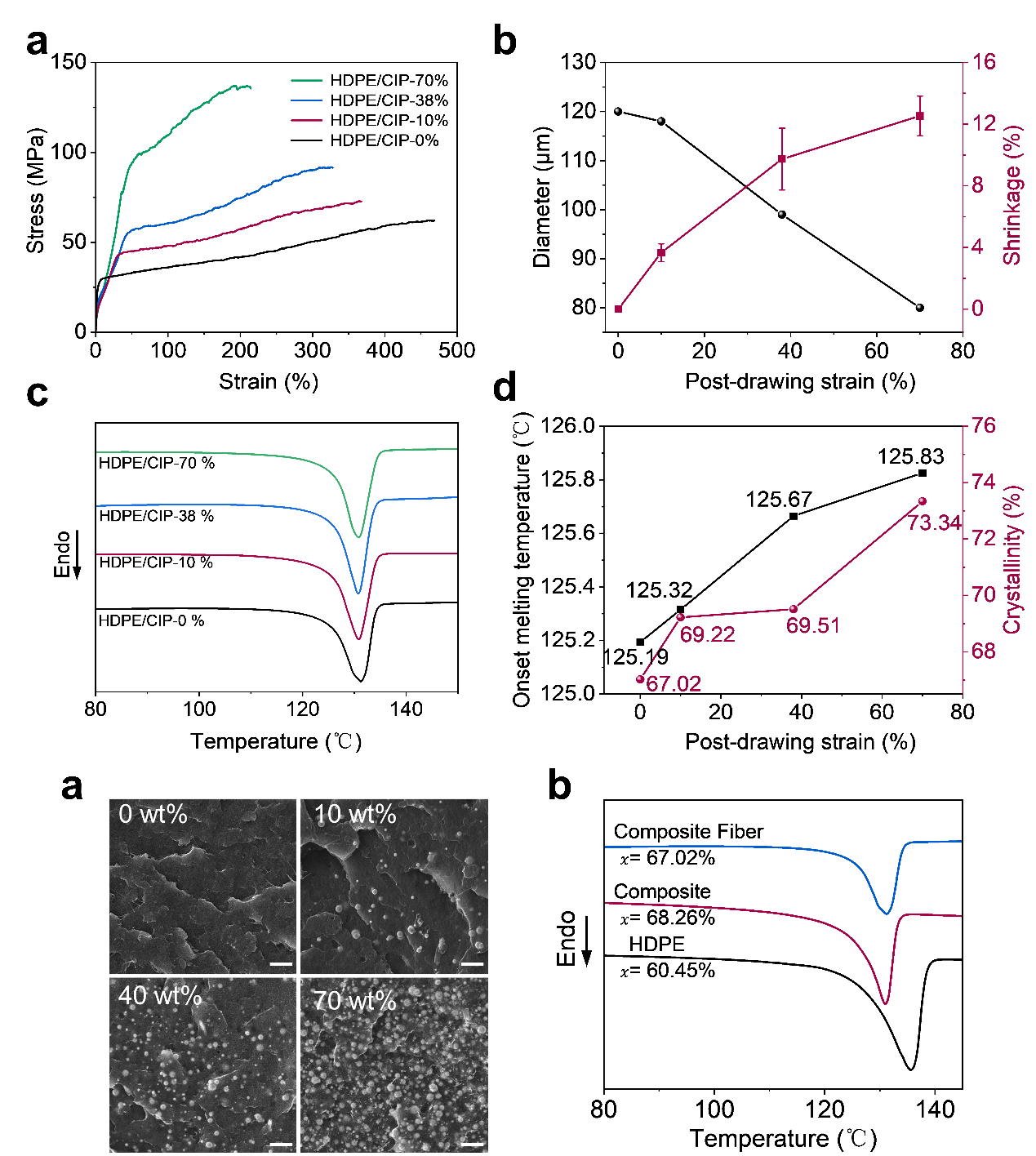


**Figure S1.** a) SEM images of fracture surfaces of the composites with CIPs contents of 0, 10, 40, 70 wt%. Scale bars, 10 µm. b) DSC curves of HDPE, bulk composite, and composite fiber with 70 wt% CIP.

**Mechanical properties of HDPE and composite bulk materials**

**Note S2**: The mechanical tests on bulk composites (15 mm $\times$10 mm$\times$0.284 mm) with different CIP weight fractions using a universal testing machine (Model 5566, Instron) at a velocity of 0.15 mm/s. Figure S2 presents the stress-strain curves along with the derived Young's modulus and elongation at break values for both HDPE and composite bulk materials.


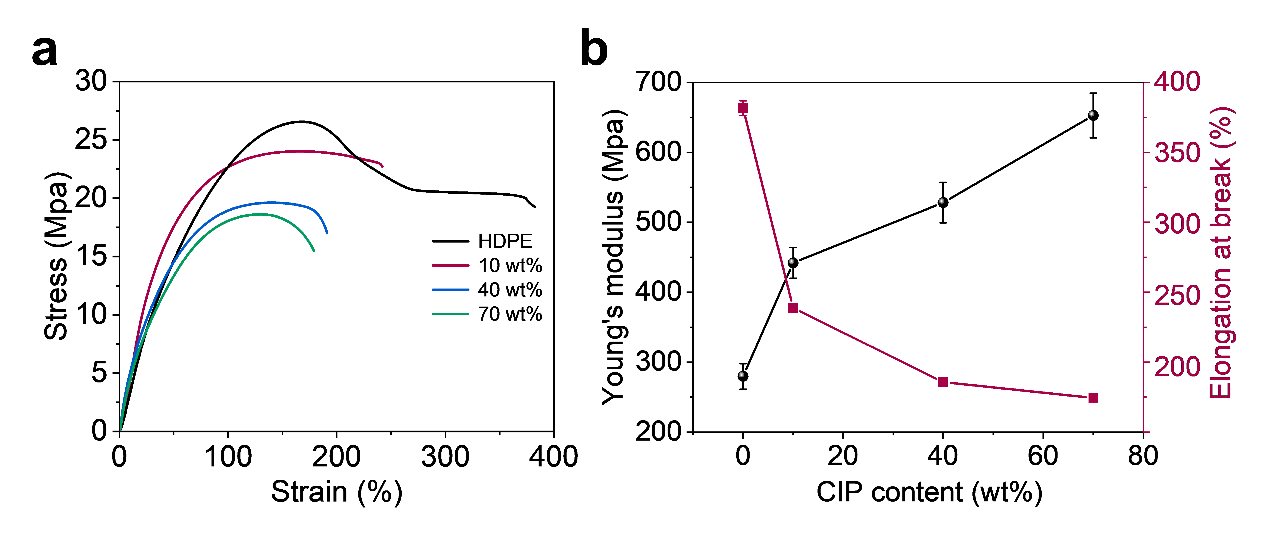


**Figure S2.** a) Stress-strain curves. b) The Young’s modulus and elongation at break values corresponding to HDPE and its composite bulk materials. Error bars represent SD, n = 3 per group.

**Abrasion resistant of magnetically active fabrics**

**Note S3**: Figure S3a shows after 1,000 abrasion cycles, the polyester embroidery blank, magnetically active fabric hinge and Miura-ori AFO all maintain their structural integrity with no observed fuzzing or yarn breakage. Figures S3b and c demonstrate the actuation properties of the magnetic fabric show minimal change even with increasing the abrasion cycles to 1,000. These results confirm the structural and functional integrity of magnetic-active fabrics with both lock-stitch and satin-stitch configurations.


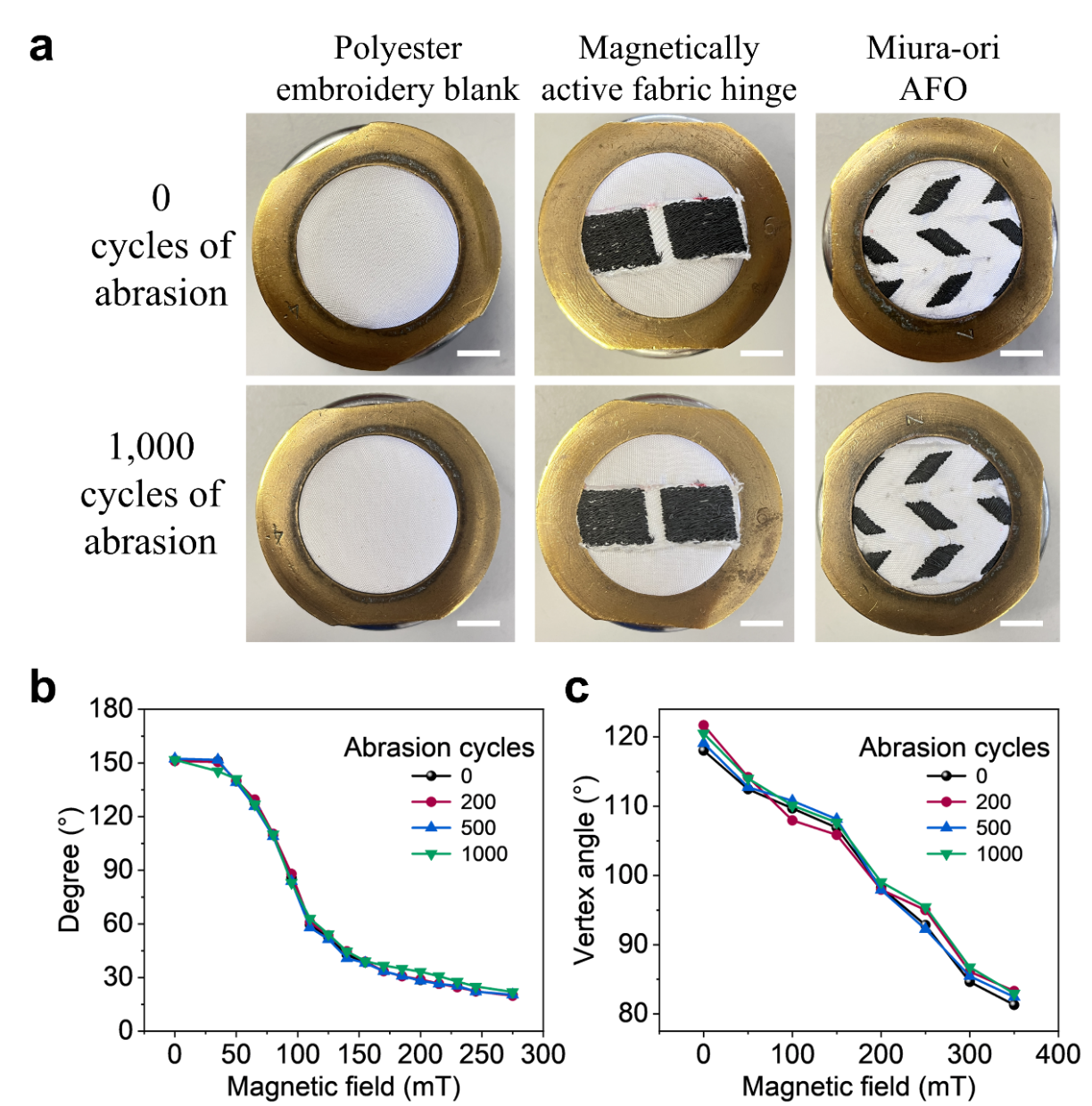


**Figure S3.** a) The photographs of polyester embroidery blank, magnetically active fabric hinge and Miura-ori AFO after 0 and 1,000 abrasion cycles. Scar bars, 10 mm. b) Hinge angle of magnetically active fabric hinge versus magnetic field strength after different abrasion cycles. c) Vertex angle ($\alpha$) of Miura-ori AFO unit cell versus magnetic field strength after different abrasion cycles.

**Water immersion resistance of magnetically active spacer fabric**

**Note S4**: We submerged the magnetically active spacer fabric in water at 20 °C and 65% relative humidity. At designated intervals (0, 1, 2, 4, and 12 h), the fabric was removed, surface water was wiped off, and it was actuated by magnetic fields to switch between standing and collapsed states. The response time of the magnetically active fabric device showed minimal variation (0.198 - 0.242 s) across all immersion durations (Figure S4), indicating negligible impact from water immersion.

**
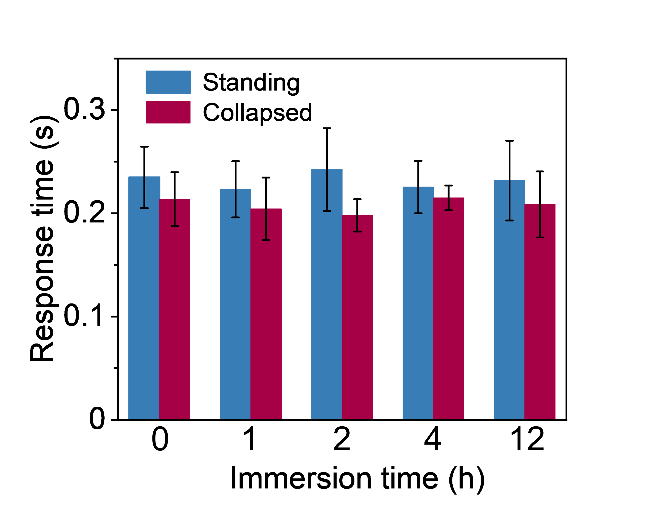
**

**Figure S4.** Response time of magnetically active spacer fabric with different water immersion time.


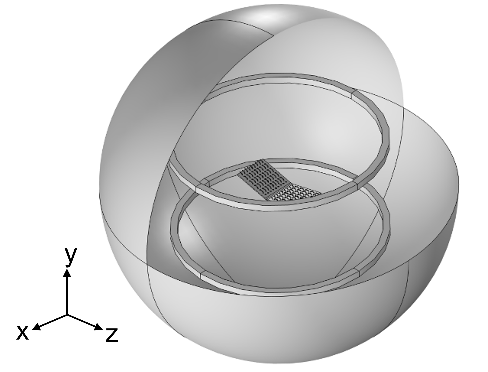


**Figure S5.** Force-magnetic coupling model


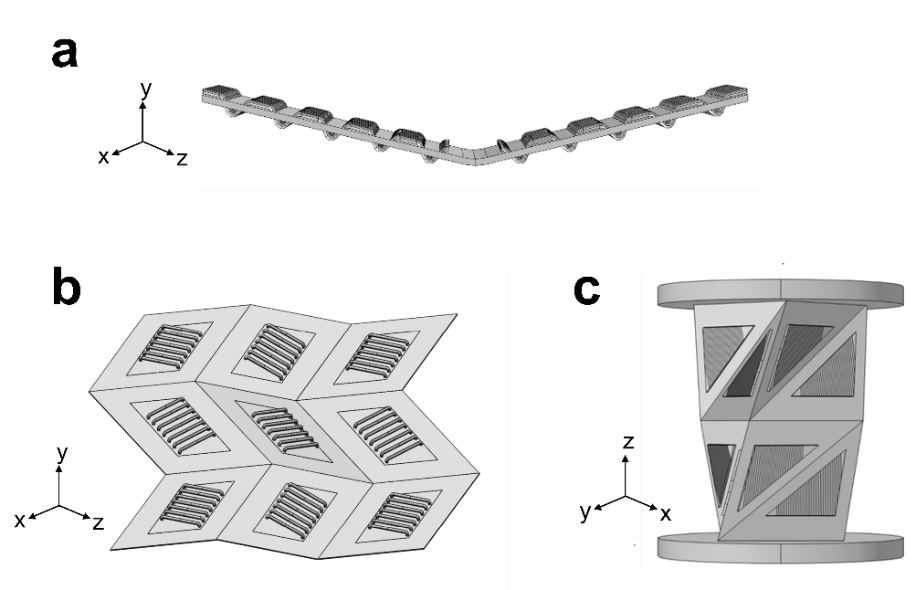


**Figure S6.** Simulation model of AFO structures. a) Magnetically active fabric hinge. b) Miura-ori AFO. c) Tube-shaped AFO with a Kresling pattern.


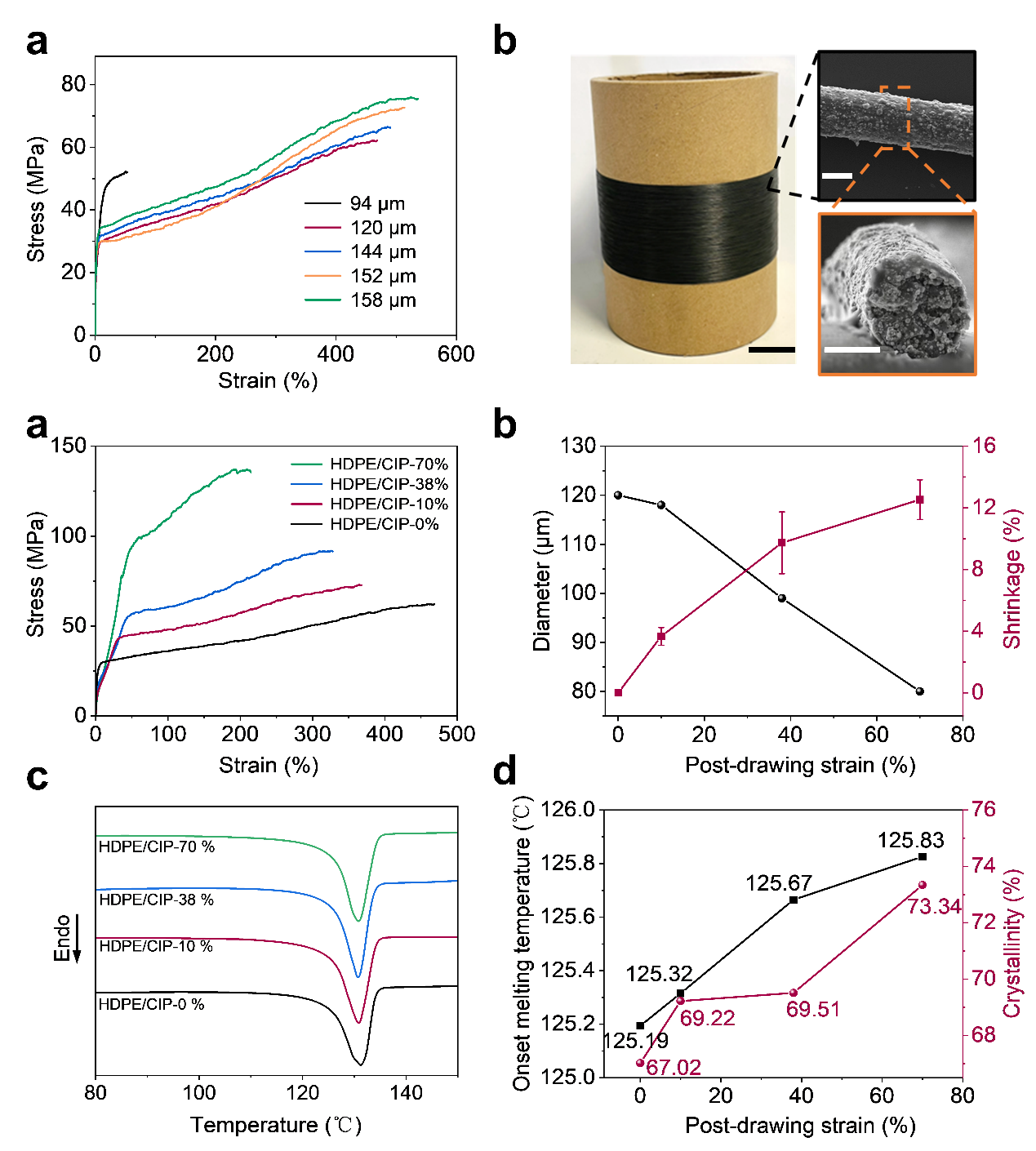


**Figure S7.** a) Stress and strain curves of as-spun magnetic filaments with different diameters. b) Photographs show a 2-km-long as-spun magnetic filament with a diameter of 120 μm wrapped on a bobbin (left), with a scale bar of 2 cm, and its microstructure (right), with scale bars of 50 μm.


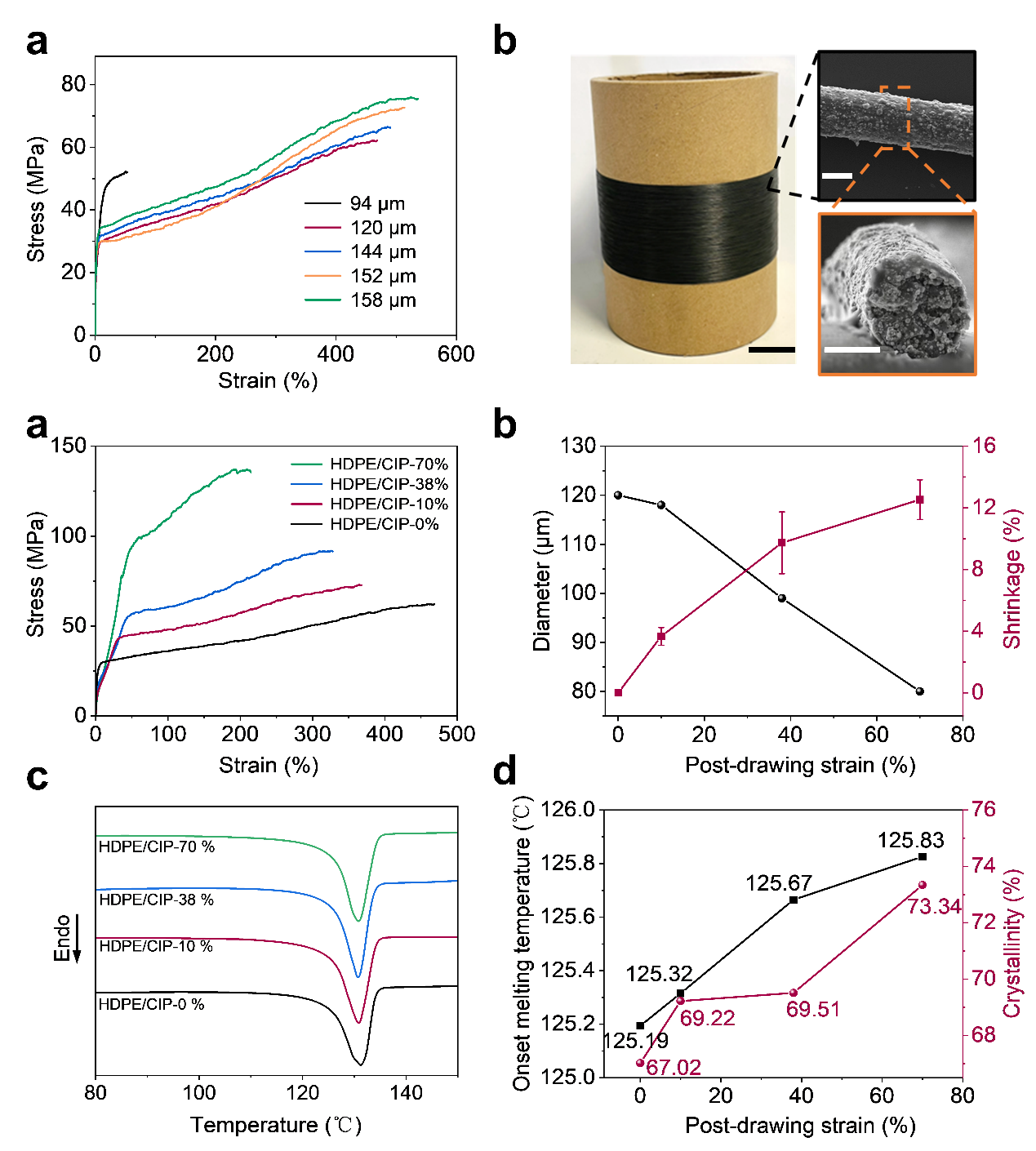


**Figure S8.** Properties of as-spun magnetic filaments with a diameter of 120 [μ](https://zh.wikipedia.org/wiki/%CE%9C)m under different post-drawing strains. a) Stress-strain curves. b) Diameter and thermal shrinkage. Error bars represent SD, n = 3 per group. c) DSC curves. d) Onset melting temperature and crystallinity.


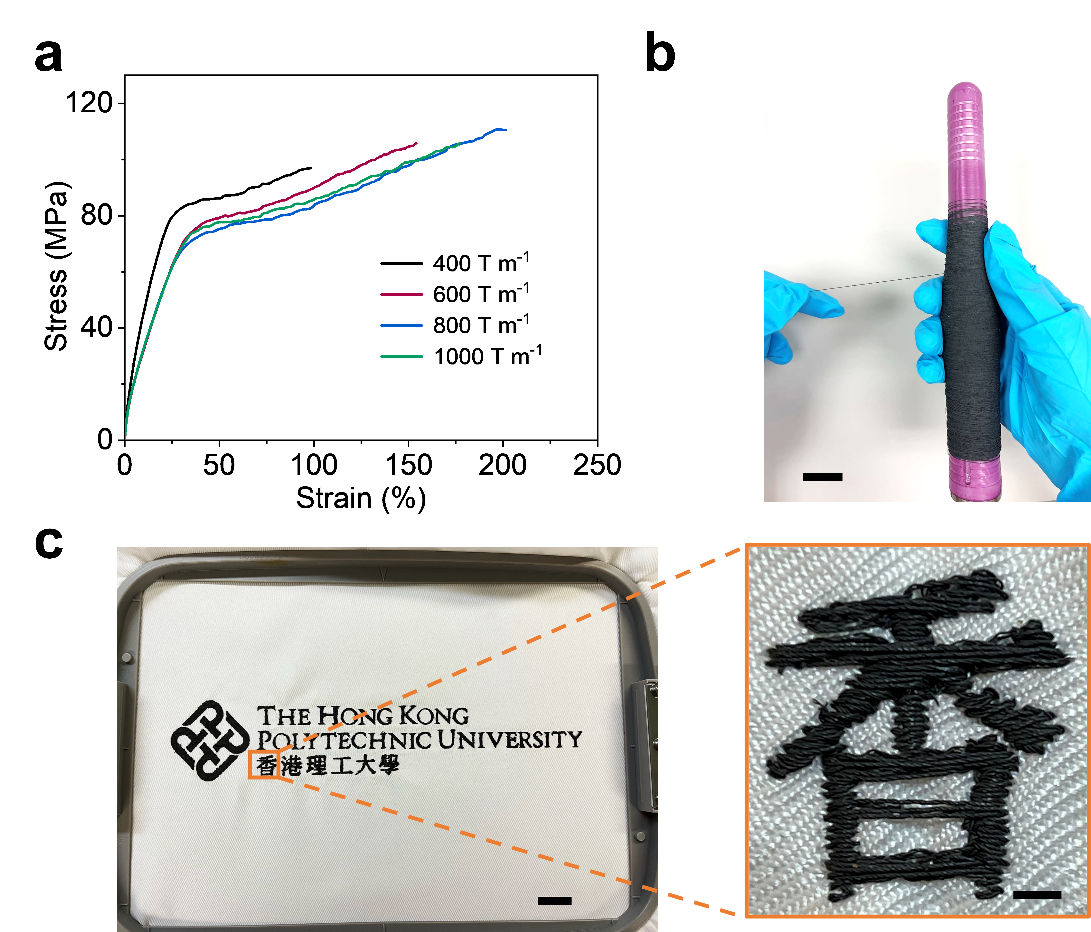


**Figure S9.** a) Stress-strain curves of magnetic yarns with varying twist levels. b) Photograph of 1.5-km-long magnetic yarns with a twist level of 800 T m^-1^. c) Photographs of an embroidery design using embroiderable magnetic yarns. Scale bars, 2 cm.


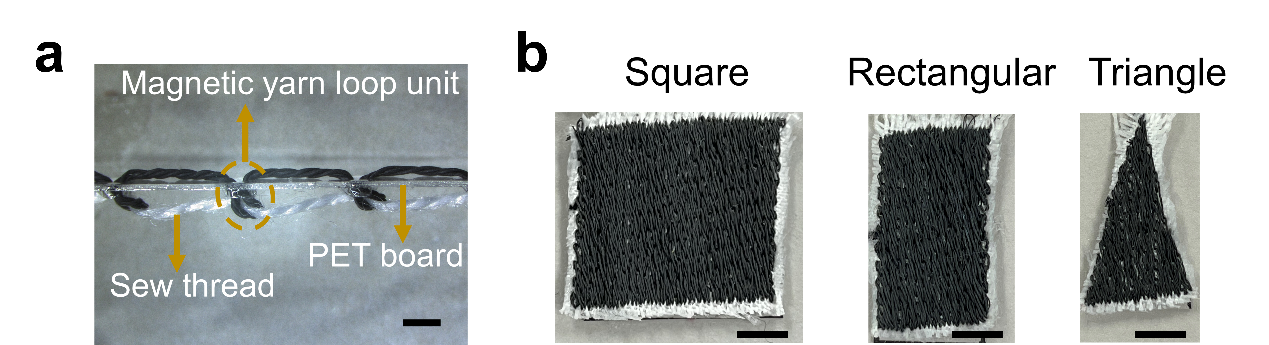


**Figure S10.** Lock-stitching embroidery structure. a) Magnetic yarn embroidered into the PET board to better display the cross-section of lock-stitching structure. Scale bars, 1 mm. b) Photographs of embroidery active fabric panels with different shapes, Scale bars, 5 mm.


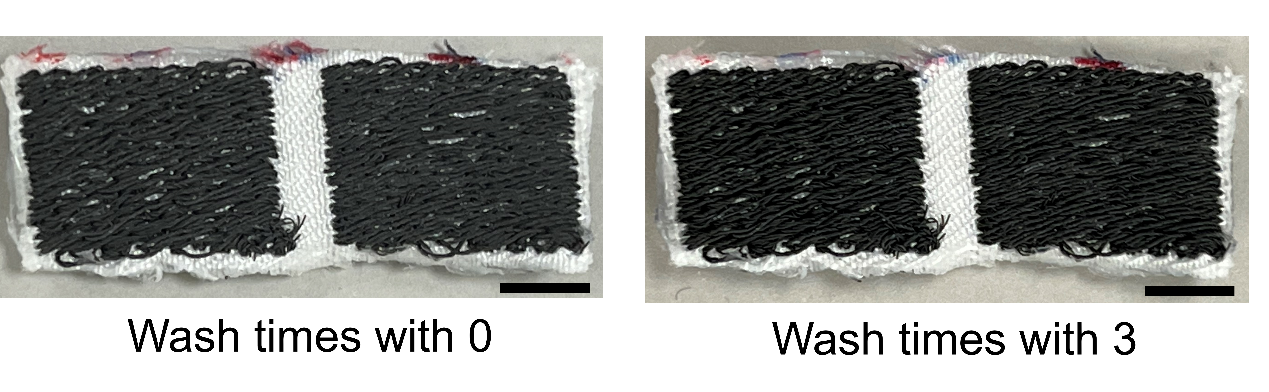


**Figure S11.** Photographs of the magnetically active fabric hinge with wash times of 0 and 3. Scale bars, 5 mm.


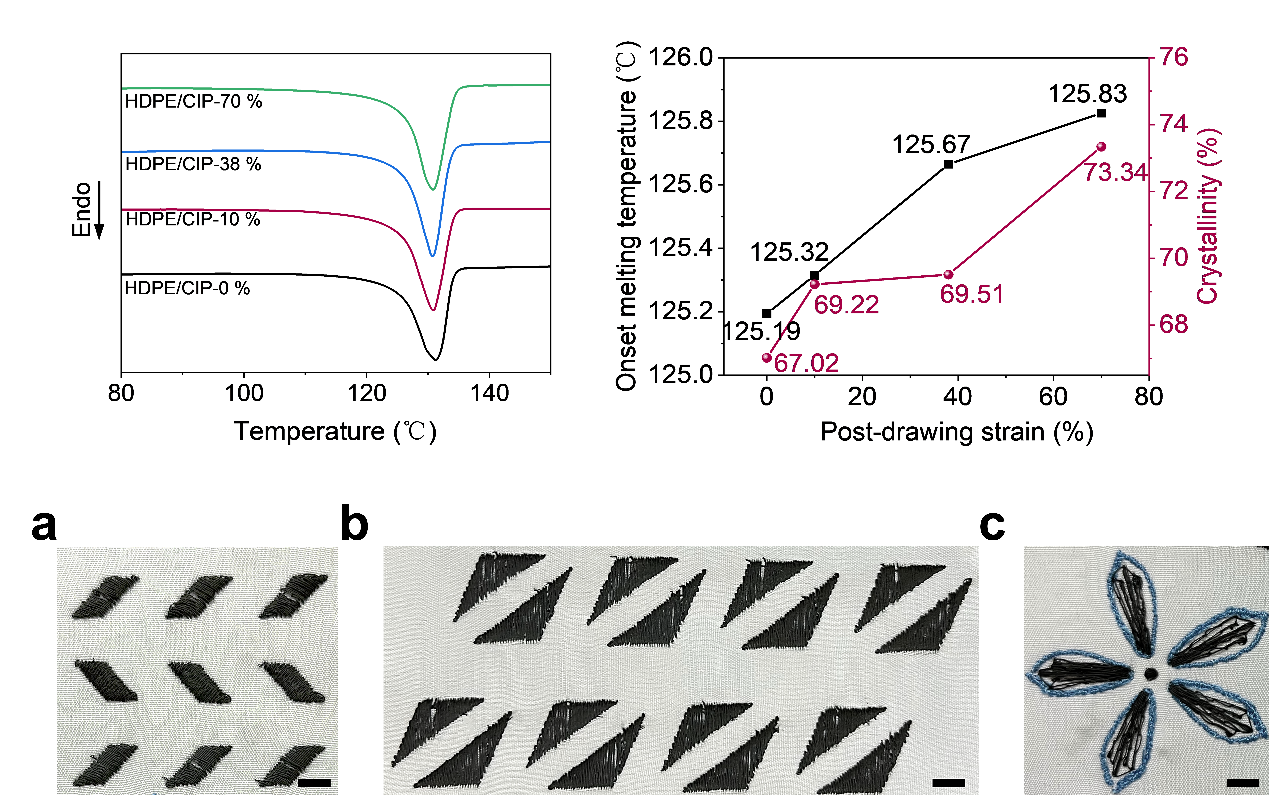


**Figure S12.** Photographs of integrating magnetic yarns into polyester fabric using satin-stitching embroidery structure. a) Miura-active fabric. b) Active fabric with Kresling pattern. c) Flower-shaped active fabric. Scale bars, 5 mm.


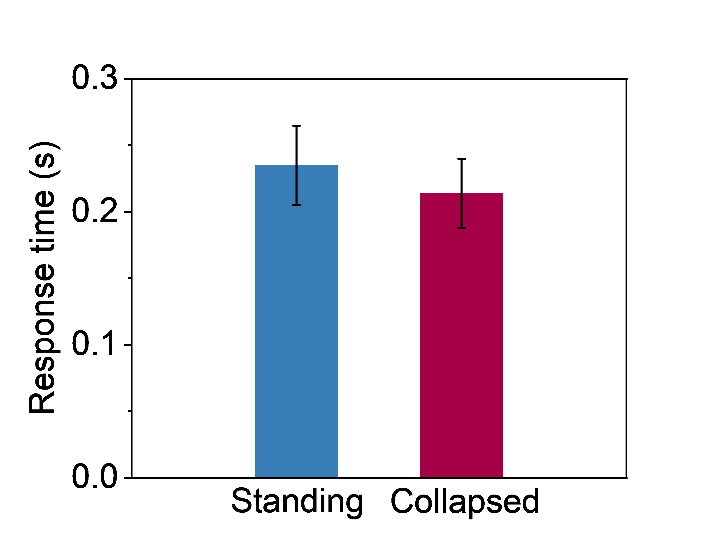


**Figure S13.** Response times (time to reach 63.2% of steady-state) of active spacer fabric switching to standing or collapsed states under a directional magnetic field of 287 mT. Error bars represent SD, n = 20 per group.


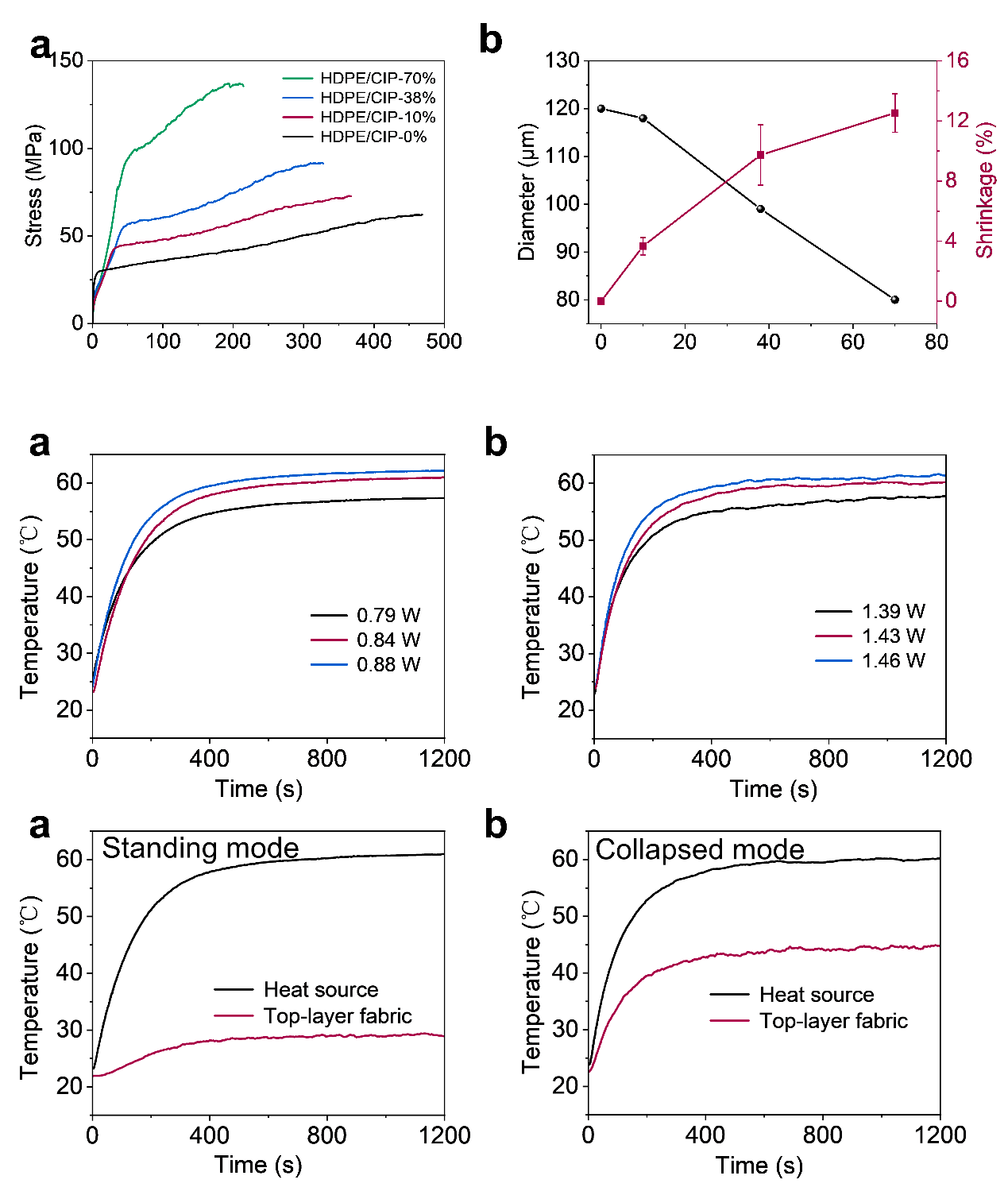


**Figure S14.** Heat sources with adjustable powers. a) Temperature and time curves of a heat source with different powers (45 mm$\times$45 mm). b) Temperature and time curves of a heat source with different powers (45 mm$\times$75 mm).


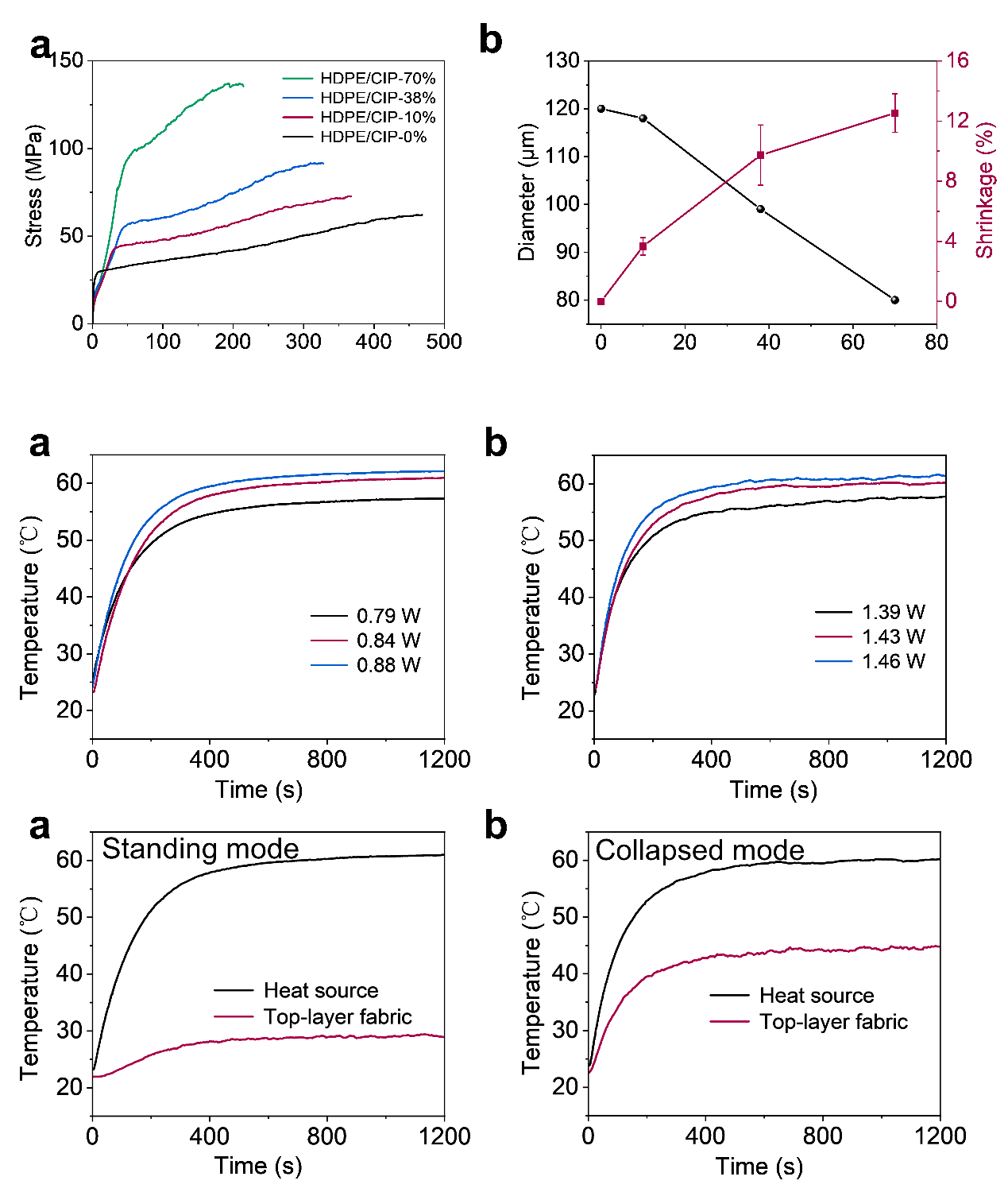


**Figure S15.** Temperature and time curves. a) Top-layer fabric temperature profiles of active spacer fabric in a standing state at 60 °C heat source. b) Top-layer fabric temperature profiles of active spacer fabric in a collapsed state at 60 ℃ heat source.

# **Supplementary Tables**

**Table S1.** The properties of material used for FEA

| Material | $\rho(kg/m^{3})$ | *E*(MPa) | $\upsilon$ | $\mu_{r}$ |
| --- | --- | --- | --- | --- |
| Polyester fabric | 2256 | 151.76 | 0.3 | - |
| Magnetic yarn | 1150 | 932.06 | 0.3 | 644.42 |

$\rho\boldsymbol{-}$ Density $E\boldsymbol{-}$ Young’s modulus $\upsilon\boldsymbol{-}$ Poisson’s ratio $\mu_{r}\mathbf{-}$ Relative permeability

**Table S2.** Performance comparison of soft-magnetic fillers for composites

| Material | *Bs* (T) | *μ_max_* (10^3^) | *Hc* (A/m) | Advantages | Limitations | Ref. |
| --- | --- | --- | --- | --- | --- | --- |
| Carbonyl Fe (this work) | 2.15 | 20 | 6 | 🞄High *B_s_*  🞄Low cost  🞄Fine particles |  | 5 |
| Fe (99.95%) | 2.15 | 230 | 4 | 🞄High *B_s_*  🞄High permeability | 🞄Expensive 🞄Oxidizes easily | 5 |
| Oriented Fe_97_Si_3_ | 2.02 | 15 - 20 | 4 - 15 | 🞄Moderate magnetic properties | 🞄Limited to sheet form 🞄Brittle | 6 |
| Fe_52_Ni_48_ | 1.6 | 100 | 4 | 🞄High permeability | 🞄Low *Bs* 🞄Expensive | 6 |
| Fe_78_B_13_Si_9_ | 1.56 | 100 | 2 - 5 | 🞄Low *Hc* | 🞄Low *Bs*  🞄Brittle  🞄Limited to ribbon forms | 6 |

**Table S3.** Comparative analysis of magnetic yarns/fibers

| Materials | Breaking stress (MPa) | Fiber/yarn diameter ($\mu$m) | Elastic modulus (Mpa) | Magnetic type | Fabrication | Application | Filler content (wt%) | Ref. |
| --- | --- | --- | --- | --- | --- | --- | --- | --- |
| Fiber (NdFeCoB/SEBS) | 13 | 300 | 3.3 | Hard magnetic | Thermal drawing | Soft robot | 20 | 7 |
| Yarn (NdFeB/PP/PTFE) | 2 | 3,850 |  | Hard magnetic | Particle flow spinning | Textile generator | 80 | 8 |
| Yarn (Nb_2_Fe_14_B/TPU) | 1.0 | 3,000 | 1.48 | Hard magnetic | Particle flow spinning | Self-powered sensor | 96 | 9 |
| Two-ply yarn (Nb_2_Fe_14_B/TPU) | 0.7 | \ | 0.7 | Hard magnetic | Particle flow spinning | Self-powered sensor | 96 | 9 |
| Three-ply yarn (Nb_2_Fe_14_B/TPU) | 0.5 | \ | 0.4 | Hard magnetic | Particle flow spinning | Self-powered sensor | 96 | 9 |
| Fiber (NdFeB/silicone polymer) | $\sim$0.8 | \ | 0.6 | Soft magnetic | Particle flow spinning | Smart textiles | 80 | 10 |
| Fiber (CIP/HDPE) | 137 | 80 | 260.3 | Soft magnetic | Melt spinning | Smart textiles | 70 | This work |
| Yarn (CIP/HDPE) | 110.5 | 320 | 382.3 | Soft magnetic | Melt spinning | Smart textiles | 70 | This work |

**Table S4.** The process parameters of the embroiderable magnetic yarns

| Process | Parameters | | Conditions | |  |
| --- | --- | --- | --- | --- | --- |
| Melt Spinning | Composite (HDPE/CIP) | | 70 wt% CIP | |  |
|  | Spinning temperature (℃) | | Hopper to die:10, 70, 150, 250, 250, 250 250, 250 | |  |
|  | Extruder speed (rpm) | | 4 | |  |
|  | Spinneret hole diameter (mm) | | 2 | |  |
|  | Distance from the spinneret to the first roller (cm) | | 107 | |  |
|  | Take-up speed (rpm) | | 130 | |  |
| Post-drawing | Hot roller temperature (℃) | | 60 | |  |
|  | Front roller speed (rpm) | | 20 | |  |
|  | Back roller speed (rpm) | | 40 | |  |
|  | Draw ratio | | 1.7 | |  |
| Twisting | Number of plies | | 4 | |  |
|  | Twist direction | | Z-twist | |  |
|  | Twist level (T m^-1^) | | 800 | |  |
| Heat-setting | Setting temperature (℃) | | 60 | |  |
|  | | Setting time (h) | | 3 | |

**Table S5.** Performance evaluation of magnetic active fabrics

| Property | Test standard | Test parameters | Results (Hinge & Miura-ori AFO) |
| --- | --- | --- | --- |
| Mechanical properties | GB/T 3923.1-2013 | Specimen: 200 🞨50 mm  Tensile Speed: 100 mm/min | Strength at break:  Warp: 483.3 N  Weft: 418.3 N  Elongation at break:  Warp: 28.7%  Weft: 29.5% |
| Abrasion resistance | ISO 12947-2:2016 | 44 mm specimen,  1,000 rubs | No fuzzing/yarn break |
| Washability | GB/T 8630-2013 | 30 min washing time | Dimensional change:$<$0.1% |

Note: The data represent two typical embroidery stitches (lock-stitching for hinge, satin-stitching for Miura-ori AFO). All tests were conducted on magnetic fabrics embroidered on polyester blanks

**Table S6.** Performance comparison of magnetic-driven fabrics and other stimulus-responsive fabrics

| Ref. | Fabric type | Stimulus | Actuator state switching time (s) | Functional Durability (Cycles) | Washability/ Water immersion | Abrasion resistance (Cycles) |
| --- | --- | --- | --- | --- | --- | --- |
| Chai., et al.^[11]^ | temperature-adaptive clothing | Thermal | 8 (bending 320°)  42 (bending 0°) | 500 | No | No |
| Roach., et al. ^[12]^ | Temperature-responsive smart shirt | Thermal | 600 (open pores)  300 (close pores) |  | Yes | No |
| Zhong., et al ^[13]^ | Humidity- Sensitive  Clothing | Humidity | 5 (flat)  7 (arched) | 400 | Yes | No |
| Li., et al.^[14]^ | Multimodal adaptive wearable | Humidity | 14 (opened 260°)  45 (0°) | 200 | No | No |
| Zhao., et al.^[15]^ | Breathable smart thermoregulation fabric | Light | 50 (opened stoma) |  | No | >5,000 |
| Guo., et al.^[16]^ | Kirigami- structure PTM device | Electrical | 6 (heating-up)  5 (cooling-down) | 500 | Yes | No |
| Hong., et al.^[17]^ | TED armband | Electrical | 120 (reach 32℃) | 1,000 | No | No |
| This work | Magnetically active fabric hinge | Magnetic | 0.334 (folding)  0.3 (opening) | $>$1,000 | Yes | >1,000 |
| This work | Magnetically active spacer fabric | Magnetic | 0.37 (standing)  0.338 (collapsed) |  | Yes | >1,000 |

**Table S7.** Energy consumption of electromagnet in actuator system

| Actuator type | Actuation frequency (Hz) | Actual powering time in each cycle (s) | Peak Current (A) | Peak Voltage (V) | Power (W) | Energy per cycle (J) |
| --- | --- | --- | --- | --- | --- | --- |
| Magnetically active fabric hinge | 1 | 0.5 | 3 | 17.1 | 51.3 | 25.65 |
| Miura-ori AFO | 0.2 | 2.5 | 5 | 28.5 | 142.5 | 356.25 |
| Tube-shaped AFO | 0.2 | 2.5 | 5 | 28.5 | 142.5 | 356.25 |
| 3D flower AFO | 0.2 | 2.5 | 3.92 | 23.6 | 92.51 | 231.28 |
| Magnetically active spacer fabric (standing/collapsed) | 0.2 | 0.708 | 3.58 | 22 | 78.76 | 55.76 |

# **Supplementary Videos**

Video S1: Automotive digital embroidery with magnetic yarns

Video S2: Actuation of magnetic yarn by magnetic torque

Video S3: Magnetically AFO with distinctive structures and deformabilities

Video S4: Magnetically active spacer fabric rapidly switches between standing and collapsed states

# **References**

1. E. Tarani, I. Arvanitidis, D. Christofilos, D. N. Bikiaris, K. Chrissafis, G. Vourlias, Calculation of the degree of crystallinity of HDPE/GNPs nanocomposites by using various experimental techniques: a comparative study. *J Mater. Sci.* **2023**, 58, 1621.
2. X. Zhang, L. Chen, Effects of laser scanning speed on surface roughness and mechanical properties of aluminum/Polylactic Acid (Al/PLA) composites parts fabricated by fused deposition modeling. *Polym. Test.* **2020**, 91, 106785.
3. K. Xu, D. Tu, T. Chen, T. Zhong, J. Lu, Effects of environmental-friendly modified rubber seed shell on the comprehensive properties of high density polyethylene/rubber seed shell composites. *Ind. Crop. Prod.* **2016**, 91, 132.
4. S. Qin, X. Lu, S.-y. Lv, W.-h. Xu, H.-h. Zhang, L.-c. Tan, J.-p. Qu, Simultaneously toughening and reinforcing high-density polyethylene via an industrial volume-pulsatile injection molding machine and Poly(ethylene terephthalate). *Compos. B: Eng.* **2020**, 198, 108243.
5. S. Tumanski, *Handbook of Magnetic Measurements*, 2016.
6. B. J. Park, F. F. Fang, H. J. Choi, Magnetorheology: materials and application. *Soft Matter*. 2010, 6, 5246.
7. H. Banerjee, A. Leber, S. Laperrousaz, R. La Polla, C. Dong, S. Mansour, X. Wan, F. Sorin, Soft Multimaterial Magnetic Fibers and Textiles. *Adv. Mater.* 2023, 35, 2212202.
8. R. Wang, Z. Du, Z. Xia, J. Liu, P. Li, Z. Wu, Y. Yue, Y. Xiang, J. Meng, D. Liu, W. Xu, X. Tao, G. Tao, B. Su, Magnetoelectrical Clothing Generator for High-Performance Transduction from Biomechanical Energy to Electricity. *Adv. Funct. Mater.* 2022, 32, 2107682.
9. J. Liu, Z. Du, Q. Wang, B. Su, Z. Xia, Particle Flow Spinning Mass-Manufactured Stretchable Magnetic Yarn for Self-Powered Mechanical Sensing. *ACS Appl. Mater. Inter.* 2022, 14, 2113.
10. X. Zhao, Y. Zhou, J. Xu, G. Chen, Y. Fang, T. Tat, X. Xiao, Y. Song, S. Li, J. Chen, Soft fibers with magnetoelasticity for wearable electronics. *Nat. Commun.* 2021, 12, 6755.
11. J. Chai, Z. Kang, Y. Yan, L. Lou, Y. Zhou, J. Fan, Thermoregulatory clothing with temperature-adaptive multimodal body heat regulation. *Cell Rep. Phys. Sci.* **2022**, 3, 100958.
12. D. J. Roach, C. Yuan, X. Kuang, V. C. Li, P. Blake, M. L. Romero, I. Hammel, K. Yu, H. J. Qi, Long Liquid Crystal Elastomer Fibers with Large Reversible Actuation Strains for Smart Textiles and Artificial Muscles. *ACS Appl. Mater. Inter.* **2019**, 11, 19514.
13. Y. Zhong, F. Zhang, M. Wang, C. J. Gardner, G. Kim, Y. Liu, J. Leng, S. Jin, R. Chen, Reversible Humidity Sensitive Clothing for Personal Thermoregulation. *Sci. Rep.* **2017**, 7, 44208.
14. X. Li, B. Ma, J. Dai, C. Sui, D. Pande, D. R. Smith, L. C. Brinson, P. C. Hsu, Metalized polyamide heterostructure as a moisture-responsive actuator for multimodal adaptive personal heat management. *Sci. Adv*. **2021**, 7, eabj7906.
15. H. Zhao, X. Qi, Y. Ma, X. Sun, X. Liu, X. Zhang, M. Tian, L. Qu, Wearable Sunlight-Triggered Bimorph Textile Actuators. *Nano Lett.* **2021**, 21, 8126.
16. Y. Guo, C. Dun, J. Xu, J. Mu, P. Li, L. Gu, C. Hou, C. A. Hewitt, Q. Zhang, Y. Li, D. L. Carroll, H. Wang, Ultrathin, Washable, and Large-Area Graphene Papers for Personal Thermal Management. *Small*. **2017**, 13, 1702645.
17. S. Hong, Y. Gu, J. K. Seo, J. Wang, P. Liu, Y. S. Meng, S. Xu, R. Chen, Wearable thermoelectrics for personalized thermoregulation. *Sci. Adv*. **2019**, 5, eaaw0536.
